# Exercise Training Transiently Increases Gut Microbiota Diversity and Short-Chain Fatty Acid Production in a Diet-Dependent Manner in Healthy Adults

**DOI:** 10.3390/microorganisms14091867

**Published:** 2026-08-22

**Authors:** Seonhong Hwang, Xuangao Wu, Jang-Won Yoon, In-Cheol Jeon, Young-In Hwang, Ki-Song Kim, Sunmin Park

**Affiliations:** 1 Department of Physical Therapy, College of Biohealth, Hoseo University, Asan-si 31499, Republic of Korea; shwang@hoseo.edu (S.H.); jyoon@hoseo.edu (J.-W.Y.); jeon6984@hoseo.edu (I.-C.J.); young123@hoseo.edu (Y.-I.H.); 2 Research Institute for Basic Sciences, Hoseo University, Asan-si 31499, Republic of Korea; 3 Smart Healthcare Convergence Research Center, Hoseo University, Asan-si 31499, Republic of Korea; 4 College of Agriculture, Yanbian University, Yanji 133002, China; niyani0@naver.com; 5 Department of Bio-Convergence System, Hoseo University, Asan-si 31499, Republic of Korea; 6 Department of Food and Nutrition, College of Biohealth, Hoseo University, Asan-si 31499, Republic of Korea

**Keywords:** exercise intervention, gut microbiome, short-chain fatty acids, bile acids, temporal dynamics, dietary modulation, causal analysis

## Abstract

Exercise alters gut microbiome composition, but the temporal dynamics and diet-dependent metabolic interactions remain unclear. We investigated longitudinal changes in gut microbiota, functional pathways, and metabolite profiles during and after an exercise intervention. Twenty-four healthy adults completed a sequential three-phase protocol: an 8-week self-directed exercise intervention, an 8-week washout, and an 8-week no-exercise control period. Fecal samples were collected at T1 (baseline), T2 (post-exercise), T3 (post-washout), and T4 (post-control). Microbiota composition was assessed by 16S rRNA sequencing, functional pathways predicted using PICRUSt2, metabolites predicted using COBRA Toolbox, and fecal SCFAs and bile acids quantified by GC and HPLC. Temporal causal relationships were examined using Tigramite analysis with dietary pattern stratification, and microbiota-environment associations were assessed by redundancy analysis (RDA). Alpha diversity was significantly higher at T2 than at T4 (*p* < 0.05). Beta diversity differed significantly between T2 and both T3 and T4, with no difference between T1 and T4, indicating reversibility. *Ruminococcus gnavus* was significantly higher at T2 than at T4 (*p* < 0.001), with several additional taxa higher at T2 at a less stringent threshold. Propionate and butyrate were elevated at T2, while total bile acids were lower. *Bacteroides thetaiotaomicron* was positively associated with body weight in the total cohort and specifically under a balanced dietary pattern (BD), with no significant time-lagged associations detected under a Western-style diet (WSD). In RDA, taxa associated with body weight substantially overlapped with taxa found to increase during exercise, whereas physical performance measures showed no direct temporal association with microbiota composition in causal analysis. In conclusion, exercise-induced changes in the gut microbiome were not sustained after structured exercise ended, suggesting that continuous exercise may be required. Diet further shaped whether microbiota–host associations were detectable, underscoring dietary pattern as a factor for future microbiome-targeted exercise interventions.

## 1. Introduction

Regular physical activity is associated with improvements in physical fitness, insulin sensitivity, and inflammatory status, and is a fundamental determinant of metabolic and cardiovascular health [1,2]. In young adults, exercise primarily enhances neuromuscular and functional performance, while changes in body composition are often modest [3]. Beyond direct physiological adaptations, growing evidence suggests that exercise influences health, in part, through interactions with the gut microbiota [4].

The gut microbiota plays a central role in energy metabolism, immune regulation, and intestinal barrier function [5]. Microbial fermentation of dietary substrates produces short-chain fatty acids (SCFAs), including acetate, propionate, and butyrate, which act as key signaling molecules regulating glucose and lipid metabolism and inflammatory pathways [6]. In addition, gut microbes convert primary bile acids into secondary bile acids, thereby shaping bile acid pools and modulating host metabolism via receptors such as the farnesoid X receptor (FXR) and the Takeda G-protein-coupled receptor 5 (TGR5) [6].

Cross-sectional studies have shown that physically active individuals and elite athletes exhibit greater gut microbial diversity and enrichment of taxa associated with SCFA production compared with sedentary controls [7]. Intervention studies further suggest that exercise can modify gut microbiota composition and metabolic output, particularly in previously sedentary individuals [8]. However, the findings remain heterogeneous, likely reflecting differences in exercise intensity, duration, dietary background, and host characteristics [8]. Importantly, most human studies have focused on short-term pre–post comparisons, and relatively few have examined microbial metabolites alongside community composition. Animal studies provide mechanistic support for exercise–microbiota interactions, demonstrating that exercise training alters gut microbial structure and that transplantation of exercise-associated microbiota improves metabolic phenotypes in recipient animals [9,10]. These observations suggest that microbial metabolites may act as mediators of exercise-induced health benefits. Nevertheless, causal inference in humans remains limited, and longitudinal data capturing both training and detraining phases are scarce. In particular, the effects of exercise on bile acid metabolism in humans have received little attention, despite the recognized role of bile acids in host–microbe crosstalk and metabolic regulation.

However, little is known about how gut microbiota and microbial metabolites respond longitudinally to individualized exercise programs under free-living conditions, particularly across both detraining and a subsequent non-exercising state. The present study aimed to investigate longitudinal changes in gut microbiota composition and fecal microbial metabolites in response to a self-directed exercise intervention in healthy young adults, using two independent post-intervention assessments to evaluate reversibility. During the exercise period, participants followed a self-directed exercise program tailored to their individual fitness level. Gut microbiota, fecal SCFAs, bile acids, body composition, physical fitness, and dietary intake were assessed at baseline (T1), after exercise (T2), after washout (T3), and after control (T4). We further examined temporal associations between microbial, dietary, and host phenotypic changes, and whether these differed by dietary pattern.

## 2. Materials and Methods

### 2.1. Participants and Study Design

This longitudinal within-subject study investigated the effects of a self-directed exercise intervention with sequential washout and control phases. Thirty-two healthy college students were voluntarily recruited, with no sex-specific inclusion or exclusion criteria applied. The final cohort included 19 males and 5 females; sex was not included as a covariate in statistical models due to the limited number of female participants, which precluded adequately powered sex-stratified analyses. Participants had no prior history of metabolic or gastrointestinal diseases and had not used antibiotics or probiotics for at least three months before enrollment (Figure S1). The study was approved by the Institutional Review Board (1041231-25-310-HR-196-02), conducted in accordance with the Declaration of Helsinki, and registered with the Korea Clinical Research Information Service (PRE20250901-004). Written informed consent was obtained from all participants.

An a priori sample size calculation was performed using G*Power (v 3.1.9.7, Dec. 25, 2025; power = 0.80, α = 0.05, medium effect size, f = 0.25), indicating that a minimum of 24 participants was required. To account for potential attrition during follow-up, 28 participants were targeted for recruitment. Ultimately, 32 participants were voluntarily enrolled between 1 October 2024 and 30 April 2025.

Participants were instructed to maintain their habitual lifestyle throughout the study, and no significant changes in these variables were observed across timepoints. No participant reported probiotic use at any timepoint, consistent with the exclusion criterion requiring no probiotic or antibiotic use within three months prior to enrollment. Lifestyle and health-related variables, including smoking status, alcohol type and amount, sleep duration, meal frequency, water intake, probiotic use, medication use, and gastrointestinal symptoms, were assessed by questionnaire at all four timepoints.

All participants completed a sequential three-phase protocol: (1) an 8-week self-directed exercise intervention period, (2) an 8-week washout period during which participants discontinued the structured exercise program and returned to habitual activity levels, and (3) an 8-week control period during which participants maintained habitual activity without any structured exercise program. During the exercise intervention period, participants independently selected their exercise type, frequency, duration, and intensity based on individual fitness goals, without a structured protocol or research-team-imposed exercise prescription. Adherence was not formally monitored by the research team but was self-monitored daily by participants and reported weekly, with goal appropriateness, goal achievement, and exercise compliance self-scored on a 0–100 scale throughout the intervention period; mean (±SD) scores were 65.0 ± 5.3, 57.4 ± 6.1, and 58.5 ± 5.7, respectively. The washout period served to separate the exercise intervention from the control period and to observe the time course of detraining, whereas the control period served as a stable within-subject reference condition reflecting habitual activity in the absence of any recent structured exercise. These two post-intervention periods allowed assessment of both the immediate trajectory of detraining (T3) and the longer-term stability of a non-exercising state (T4), relative to the pre-exercise baseline (T1). Eight participants were excluded due to incomplete physical performance testing (*n* = 5) or missing fecal samples (*n* = 3), resulting in a final analytical cohort of 24 participants with complete data across all four time points. No adverse events were reported during the study.

### 2.2. Clinical and Demographic Assessment

Comprehensive demographic data were collected, including age, gender, and BMI. Skeletal muscle mass and body fat mass were measured using bioelectrical impedance analysis (InBody 270, InBody Co., Seoul, Republic of Korea). SBP and DBP were measured using an electronic sphygmomanometer (SELVAS Healthcare, Daejeon, Republic of Korea).

### 2.3. Physical Performance and Dietary Intake Measurements

Physical performance was assessed at all four time points using standardized protocols. Grip strength was measured using a Jamar Plus+ Digital Hand Dynamometer (Performance Health, Warrenville, IL, USA), with the maximum value from three trials per hand recorded. Standing long jump distance was measured from the starting line to the nearest heel contact, with the longest of three attempts recorded. Vertical jump height was measured using a tape ruler mounted on a wall, with participants marking their maximum reach height while standing and their peak jump height, with the difference representing jump height (best of three trials recorded). One-leg standing balance (OLS) was measured as the time participants could maintain a stable single-leg stance with eyes open and hands on their hips, with the longest of three trials recorded.

Dietary intake was assessed at all four time points using a validated 106-item Semi-Quantitative Food Frequency Questionnaire (SQFFQ) used in the Korean Genome and Epidemiology Study (KoGES) [11]. Intake frequencies were converted to daily frequencies and multiplied by standard portion sizes (g/serving) to calculate average daily intake for each food item (g/day). The 106 food items were aggregated into 23 food groups based on nutritional similarity. Principal component analysis (PCA) with varimax rotation was performed to identify major dietary patterns. Dietary patterns were extracted using eigenvalues >1.5, and food groups with factor loadings ≥0.4 were considered significant contributors to each pattern [12,13]. Two major dietary patterns were identified: (1) a BD characterized by high intake of vegetables, fruits, whole grains, and fish; and (2) a WSD characterized by high intake of meats, added fats, fast food, and sugar-sweetened beverages. Participants were categorized into the WSD (*n* = 7) and BD (*n* = 17) groups based on the median split of the WSD scores. Total energy intake and macronutrient composition (carbohydrate, protein, fat) were calculated using the web-based CAN-Pro system (version 5.0, Korean Nutrition Society, Seoul, Republic of Korea). Relative dietary characteristics were obtained by dividing the SQFFQ frequency values of each participant by their sum. Principal coordinate analysis was used to visualize the Bray–Curtis distance. Repeated measures PERMANOVA (9999 intra-participant permutations) was used to assess temporal differences, followed by Benjamini–Hochberg corrected pairwise comparisons. PERMDISP was used to assess multivariate dispersion.

### 2.4. Fecal Sample Collection and SCFA and Bile Acid Measurement

Fecal samples were collected by participants at home at the four designated time points, immediately stored at −20 °C, and subsequently transferred to −80 °C for long-term storage. Samples were freeze-dried, and 0.2 g dry weight was used for SCFA and bile acid measurements to normalize for variable fecal water content.

For SCFA analysis, freeze-dried fecal samples were mixed with ethanol, and the pH was adjusted to 2–3 with hydrochloric acid (HCl). Samples were centrifuged (10,000× *g*, 15 min, 4 °C) and filtered through 0.45 µm membranes. SCFAs (acetate, propionate, and butyrate) were measured using an Agilent Technologies 6890N gas chromatograph equipped with a high-polarity phase column (HP-FFAP, 19091F-433E, Agilent Technologies, Santa Clara, CA, USA). The injection port temperature was set to 220 °C, and the flame ionization detector (FID) temperature was set to 250 °C. The oven temperature was started at 50 °C and increased to 200 °C at a rate of 15 °C/min, with a 3 min hold, for a total run time of 10 min. Acetate, propionate, and butyrate standards (Sigma-Aldrich, SBR00030, St. Louis, MO, USA) were prepared at 10 mM, 5 mM, and 2 mM to generate standard curves for quantification. SCFA concentrations were expressed as amounts per gram of dry fecal weight.

For bile acid analysis, freeze-dried fecal samples were mixed with methanol, centrifuged (10,000× *g*, 15 min, 4 °C), and filtered through 0.45 µm membranes. Bile acids were analyzed using an Agilent 1100 HPLC system (Agilent Technologies, Waldbronn, Germany) equipped with a SunFire C18 column (186002560, Waters, Milford, MA, USA) and an evaporative light scattering detector (ELSD). The mobile phase consisted of acetonitrile and 0.3% (*v*/*v*) formic acid in water, at a flow rate of 1 mL/min, with a gradient of 56:44 (acetonitrile: aqueous formic acid) from 0–7 min, changing to 95:5 from 18–25 min. The column temperature was maintained at 30 °C, and the injection volume was 30 µL. Bile acid standards, including cholic acid (CA), chenodeoxycholic acid (CDCA), ursodeoxycholic acid (UDCA), lithocholic acid (LCA), and deoxycholic acid (DCA) (Sigma-Aldrich, St. Louis, MO, USA), were used to generate calibration curves. Results were expressed as concentrations per gram of dry fecal weight.

### 2.5. Assessment of Fecal Bacterial Composition

Total genomic DNA was extracted from fecal samples using the QIAamp PowerFecal Pro DNA Kit (QIAGEN, Venlo, The Netherlands). The V3–V4 hypervariable regions of the bacterial 16S rRNA gene were amplified using universal primers 341F (5′-CCTACGGGNGGCWGCAG-3′) and 805R (5′-GACTACHVGGGTATCTAATCC-3′). Polymerase chain reaction (PCR) products were purified and recovered using AMPure XP magnetic beads (Beckman Coulter, Brea, CA, USA). Paired-end sequencing was performed on the Illumina MiSeq platform (Illumina, San Diego, CA, USA).

Raw sequence data were processed using Quantitative Insights Into Microbial Ecology (QIIME2, v2022.2). Primer-trimmed sequences were denoised using the Divisive Amplicon Denoising Algorithm (DADA2) [14], with forward reads trimmed at 12 bp and truncated to 260 bp, and reverse reads trimmed at 8 bp and truncated to 265 bp, generating amplicon sequence variants (ASVs) using default parameters. Taxonomic assignment was performed using the Greengenes2 database (version 2024.09) [15]. ASVs were phylogenetically placed into the Greengenes2 full-length 16S rRNA backbone tree using the q2-greengenes2 plugin, and taxonomic lineages were derived from phylogenetic positions using the taxonomy-from-table command. ASVs sharing identical taxonomic assignment were merged to construct an operational taxonomic unit (OTU) table for downstream analysis. The resulting OTU table was filtered to retain only OTUs present in at least 10% of samples with an average relative abundance ≥0.01. All taxa reported in subsequent analyses therefore refer to these taxonomy-merged OTUs rather than individual ASVs.

### 2.6. Downstream Metagenomic Analysis

Phylogenetic diversity was calculated using the align-to-tree-mafft-fasttree pipeline in QIIME2. Alpha diversity was assessed using the Shannon index via the core-metrics-phylogenetic workflow. Beta diversity was evaluated using unweighted UniFrac distance matrices and visualized through principal coordinates analysis (PCoA).

Functional pathway predictions were performed using PICRUSt2 (v 2.1.2; Feb. 20, 2026) to infer the metabolic potential of the gut microbiome from 16S rRNA gene sequences. Beta diversity was assessed using PERMANOVA (999 permutations) on unweighted UniFrac distances, with pairwise comparisons between timepoints FDR-corrected (*q* < 0.1). To further evaluate compositional reversibility, pairwise Bray–Curtis dissimilarity was additionally assessed using PERMANOVA for all timepoint comparisons. For both metrics, pairwise comparisons were conducted between the two timepoints.

Metabolite predictions were performed using personalized microbial community models generated with the Agora2 (version 2) resource within the COBRA Toolbox v. 3.6 (March 15, 2026). Strain-level reconstructions were aggregated into species-level pan-models using the createPanModels function. Individual models were constructed via the initMgPipe pipeline v. 2.0 (April 20, 2026) by integrating species-level relative abundance profiles. Dietary constraints were applied based on the average Caucasian diet model from the Virtual Metabolic Human (VMH) database (http://vmh.life April 10, 2026). Community biomass reactions were defined using species abundances as stoichiometric coefficients, with growth rates bounded by experimental constraints. All models were solved in MATLAB R2021b using the IBM CPLEX 12.10 solver (Armonk, New York, USA). Differential abundance of predicted pathways and metabolites between time points was assessed with the Benjamini–Hochberg FDR correction. Results were visualized using volcano plots with effect sizes represented as log2 fold changes and significance indicated by −log10(*q*-value).

RDA was performed to explore the relationships between gut microbiota composition and environmental variables (dietary intake and physical performance measures), as well as metabolic outputs (SCFAs and bile acids), using the vegan package (999 permutations; *p* < 0.05). RDA ordination biplots were generated to visualize the associations between bacterial OTUs and explanatory variables, with the length and direction of variable vectors indicating the strength and direction of their correlations with microbial community composition.

Spearman’s rank correlation analysis was performed using the SciPy function spearmanr (version 1.16.1) to assess relationships among bacterial species, including significant temporal changes, predicted metabolites, body composition indicators, and dietary intake.

### 2.7. Tigramite Longitudinal Causal Analysis

To identify potential causal relationships among gut microbiota, dietary intake, and host metabolic phenotypes, temporal causal analysis was performed using the Tigramite (5.2.8.2) Python v. 3.14.7 (May 7, 2026) package [16]. Subjects with missing values at any time point were excluded. Clinical indicators, dietary intake, and metabolic variables were Z-score normalized using StandardScaler from scikit-learn (1.7.2), while microbiome species relative abundance data underwent centered log-ratio (CLR) transformation. The Peter and Clark Momentary Conditional Independence (PCMCI)+ algorithm was applied to infer lagged and contemporaneous causal links while controlling for autocorrelation. Data were structured into a longitudinal three-dimensional array across four time points. Partial correlation was used as the conditional independence test, assuming linear dependencies. The maximum time lag was set to 1, classifying causal links into contemporaneous effects (Lag 0) and time-lagged effects (Lag 1).

### 2.8. Statistical Analysis

Anthropometric measures, physical performance, α-diversity indices, and metabolite concentrations (SCFAs and bile acids) were analyzed using linear mixed-effects models, with time point as a fixed effect and participant as a random effect to account for repeated measurements. Post hoc pairwise comparisons between time points were adjusted using the false discovery rate (FDR), with *q* < 0.05 considered statistically significant. Differential abundance analysis of bacterial taxa was performed using MaaSlin2 (v1.22.0) with linear mixed-effects models, including time point as a fixed effect and participant as a random effect; taxa were considered differentially abundant at nominal *p* < 0.001, given the exploratory nature of this analysis and the large number of taxa tested. Functional pathway predictions (PICRUSt2) and metabolite predictions (COBRA Toolbox) were similarly analyzed using linear mixed-effects models, with differential abundance assessed at FDR-adjusted *q* < 0.1.

## 3. Results

### 3.1. Participant Characteristics and Exercise Response

Twenty-four participants (19 males, 5 females; mean age 22.67 ± 1.63 years; BMI 23.4 ± 2.57 kg/m^2^) completed all four phases of the trial. Participant characteristics at the T1 and T3 time points are presented as baseline values for the exercise and control trials, respectively (Table S1). The participants had normal SBP and DBP at T1 and T3 time points (Table S1). Lifestyle and health-related variables, including smoking status, alcohol type and amount, sleep duration, meal frequency, water intake, probiotic use, medication use, and gastrointestinal symptoms, were not significantly different across time points. Dietary composition, assessed via PERMANOVA on Bray–Curtis distances of relative SQFFQ food-group frequencies, differed significantly across time points. Pairwise comparisons showed significant differences between T1 and T2, T1 and T3, and T1 and T4 (*q* = 0.0012 for all three; Figure S2), with participants reporting proportionally higher intake at T1 than at subsequent timepoints. No significant differences were observed between T2, T3, and T4 (*q* ≥ 0.865). Multivariate dispersion did not differ significantly between timepoints (PERMDISP, all *q* ≥ 0.25), indicating that the PERMANOVA result reflects a true difference in dietary composition (centroid location) rather than differences in within-group variability. This shift in relative dietary composition occurred despite the absence of a statistically significant difference in total self-reported energy intake across timepoints, likely reflecting high variability in absolute energy intake relative to a more consistent shift in relative dietary composition.

Effects of the exercise intervention on body composition and physical performance have been reported previously using repeated-measure analysis [17]. Among the longitudinal changes across the four sequential time points, body weight showed no significant change at T2 compared to T1, but was significantly lower at T3 and T4 than at T1 (Figure S3A). Body fat mass was significantly lower at T2, T3, and T4 compared to T1 (Figure S3B). Visceral fat percentage was significantly lower at T2 and T3 compared to T1 (Figure S3C). Grip strength showed a non-significant increasing trend at T2 compared to T1, followed by a significant decline thereafter, resulting in significantly lower values than T1 at T3 and T4 (*q* < 0.05) and significantly lower values than T2 (*q* < 0.001; Figure S4A). In physical performance, long jump and vertical jump were significantly increased at T2 compared to T1 (*q* < 0.05), declined significantly from T2 to T3 (*q* < 0.001), and remained significantly different from T1 at T4 (*q* < 0.001), indicating that exercise-induced improvements were not fully sustained but did not fully revert to baseline levels (Figure S4B,C). OLS followed a pattern similar to long jump and vertical jump, with significant differences observed across all pairwise comparisons (*q* < 0.05–0.001; Figure S4D).

### 3.2. Microbial Diversity and Changes in Gut Microbiome Composition

**Figure 1:**
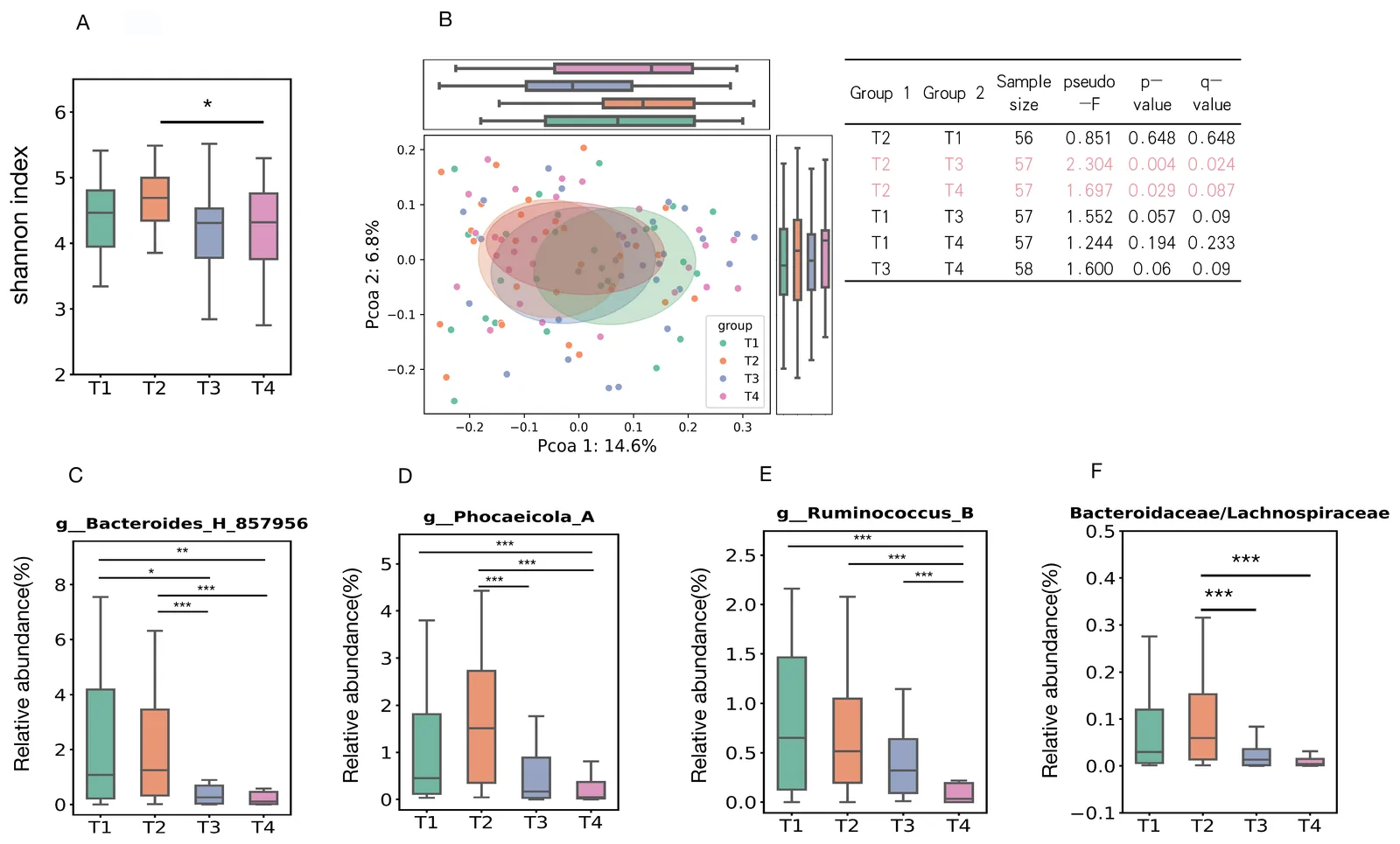
Microbial characteristics across the different time points. (**A**) Shannon diversity index (alpha diversity) measured at baseline (T1), after 8-week exercise intervention (T2), after an 8-week washout (T3), and after an 8-week control period (T4). (**B**) Principal coordinates analysis (PCoA) of beta diversity based on unweighted UniFrac distances with pairwise comparisons between time points FDR-corrected (*q* < 0.1). (**C**–**E**) Relative abundance of key bacterial taxa: (**C**) *Bacteroides xylanisolvens*, (**D**) *Phocaeicola vulgatus*, and (**E**) *Ruminococcus gnavus*. (**F**) Ratio of abundance of Bacteroidaceae to Lachnospiraceae family. Box plots show the median (center line), interquartile range (box), and range (whiskers). Horizontal lines with asterisks indicate significant pairwise comparisons. * False discovery rate (FDR)-adjusted *q* < 0.05, ** *q* < 0.01, *** *q* < 0.001. (linear mixed-effects models with post hoc pairwise comparisons for panels (**A**,**C**–**F**); PERMANOVA for panel (**B**)). *n* = 24 participants.

Gut microbial alpha diversity (Shannon index) was highest at T2 (post-exercise), with a marginal difference compared to T1 (*q* = 0.092), and was significantly greater than at T4 (*q* < 0.05) but did not differ significantly from T3 (Figure 1A). Beta-diversity analysis based on unweighted UniFrac distances revealed significant differences in overall microbial community structure between T2 and T3 (PERMANOVA, *p* = 0.004, *q* = 0.024) and between T2 and T4 (*p* = 0.029, *q* = 0.087; Figure 1B). No significant differences were observed between T1 and T2 or between T1 and T4. To further assess compositional reversibility, pairwise Bray–Curtis dissimilarity was additionally evaluated using PERMANOVA. No significant differences were observed for any pairwise timepoint comparison (all *q* ≥ 0.63; Figure S5).

Cumulative genus-level composition across the four timepoints is shown in Figure S6. Overall community composition remained broadly stable across timepoints, with *Blautia*_A, Bifidobacterium, and Collinsella consistently representing the most abundant genera at all four timepoints. Three genera showed significant temporal changes. *Bacteroides*_H abundance did not differ significantly between T1 and T2, but was significantly higher at T1 than at T3 and T4, and higher at T2 than at T3 (Figure 1C). *Phocaeicola*_A abundance did not differ significantly between T1 and T2 or between T1 and T3, but was significantly higher at T1 than at T4, and higher at T2 than at both T3 and T4 (Figure 1D). *Ruminococcus*_B abundance did not differ significantly among T1, T2, and T3, but was significantly higher at T1, T2, and T3 than at T4 (Figure 1E). The Bacteroidaceae/Lachnospiraceae ratio was significantly higher at T2 than at both T3 and T4 (Figure 1F).

**Figure 2:**
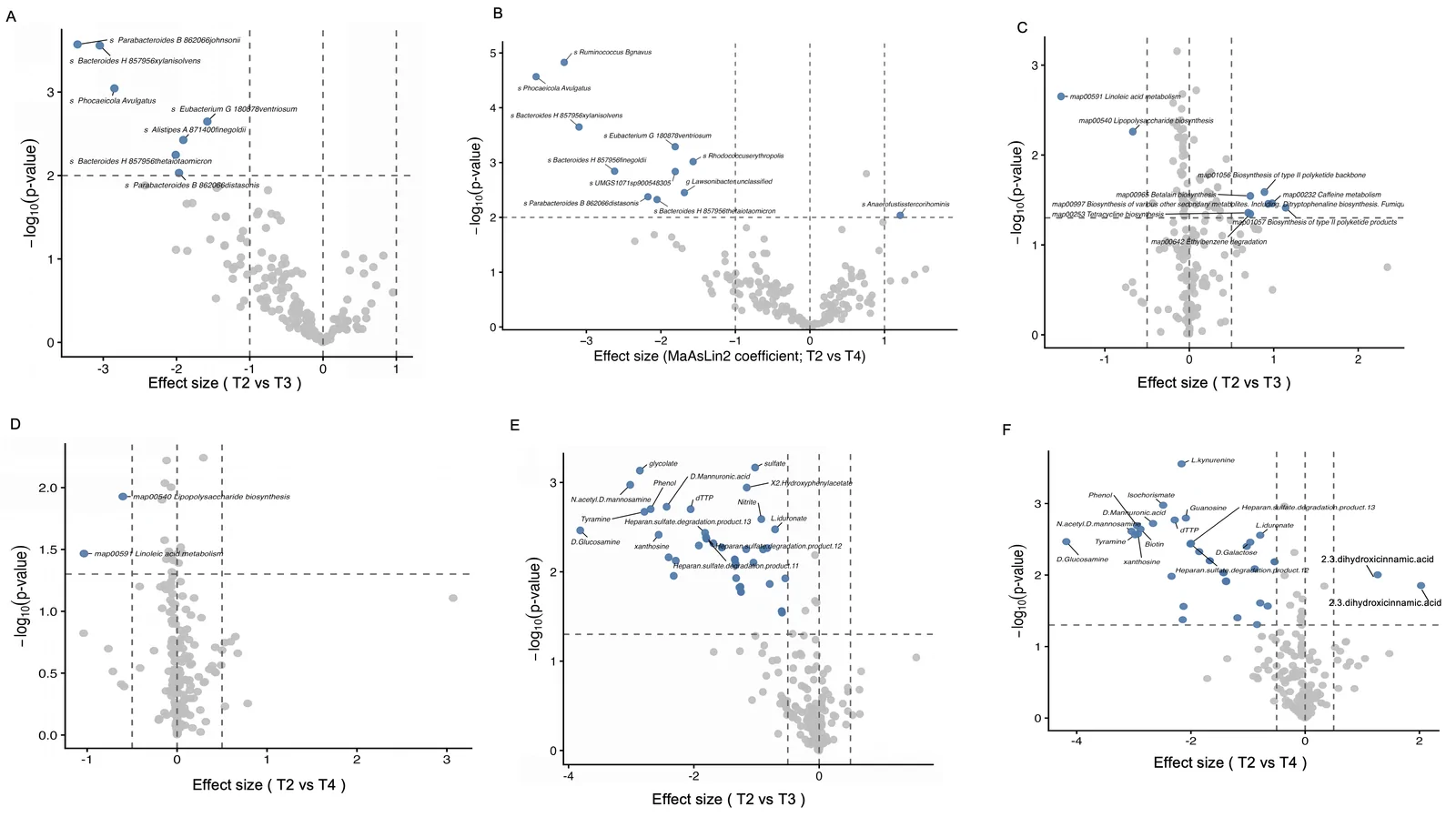
Differential abundance of bacterial taxa, functional pathways, and predicted metabolites. Volcano plot showing differential abundance of bacterial species (**A**) between T2 (exercise) and T3 (washout) and (**B**) between T2 (exercise) and T4 (control). Each point represents a bacterial species. Differential abundance of predicted functional pathways (Phylogenetic Investigation of Communities by Reconstruction of Unobserved States 2 [PICRUSt2]) (**C**) between T2 and T3 and (**D**) between T2 and T4. Each point represents a Kyoto Encyclopedia of Genes and Genomes (KEGG) pathway. Differential abundance of predicted metabolites (COnstraint-Based Reconstruction and Analysis [COBRA] Toolbox) (**E**) between T2 and T3 and (**F**) between T2 and T4. Each point represents a predicted metabolite. The *X*-axis shows log2 fold change (negative values indicate lower abundance at T2; positive values indicate higher abundance at T2). The *y*-axis shows −log10(*q*-value). Vertical dashed lines indicate log2 fold change thresholds of ±1. Horizontal dashed lines indicate false discovery rate (FDR)-adjusted significance thresholds. Colored/labeled points represent significantly differentially abundant features. Analysis performed using MaaSlin2 (v1.22.0) for bacterial taxa and linear mixed-effects models for pathway and metabolite predictions. *n* = 24 participants.

Volcano plot analysis identified bacterial species differentially abundant between post-exercise (T2) and subsequent time points (Figure 2 and Figure S7). In the T2 versus T3 comparison, *Parabacteroides johnsonii* and *Bacteroides xylanisolvens* showed the largest effect sizes (*q* < 0.001), with *Phocaeicola vulgatus*, *Eubacterium ventriosum*, *Alistipes finegoldii*, and *Bacteroides thetaiotaomicron* also significantly higher at T2 than at T3 (Figure 2A). In the T2 versus T4 comparison, *Ruminococcus gnavus*, *Phocaeicola vulgatus*, and *Bacteroides xylanisolvens* were significantly higher at T2 than at T4 (*q* < 0.001), whereas *Anaerofustis stercorihominis* was significantly higher at T4 than at T2 (Figure 2B). Most differentially abundant taxa identified were thus increased during exercise (T2) relative to washout (T3) and control (T4), with *Anaerofustis stercorihominis* as a notable exception showing reduced abundance during exercise.

### 3.3. Pathway-Level Differences by PICRUSt2

When T2 and T3 were compared, linoleic acid metabolism (map00591) and lipopolysaccharide (LPS) biosynthesis (map00540) were significantly enriched at T2, with large negative effect sizes (*p* < 0.01), indicating markedly higher abundances at T2 than at T3 (Figure 2C). Conversely, caffeine metabolism (map00232) and several other pathways—including biosynthesis of various secondary metabolites, betalain, tetracycline, and type II polyketide products—showed significant enrichment at T3 relative to T2 (positive effect sizes). In the T2 versus T4 comparison, LPS biosynthesis and linoleic acid metabolism again exhibited significant enrichment at T2, with negative effect sizes (*p* < 0.05), though the magnitude of the difference was smaller than in the T2 vs. T3 comparison (Figure 2D). LPS biosynthesis and linoleic acid metabolism were thus consistently elevated during the exercise period (T2) relative to both post-washout (T3) and post-control (T4) periods.

### 3.4. Metabolite-Level Differences by COnstraint-Based Reconstruction and Analysis (COBRA) Toolbox

When T2 was compared with T3, glycolate, sulfate, phenol, and D-mannuronic acid exhibited large negative effect sizes with high statistical significance (*p* < 0.001), indicating markedly higher abundances at T2 than at T3 (Figure 2E). Additional significantly altered metabolites included N-acetyl-D-mannosamine, tyramine, D-glucosamine, deoxythymidine triphosphate (dTTP), and multiple heparan sulfate degradation products. In the T2 versus T4 comparison, L-kynurenine exhibited the largest negative effect size and highest statistical significance (*p* < 0.001), indicating a markedly higher abundance at T2 than at T4 (Figure 2F). Additional metabolites showing significantly higher levels at T2 included phenol, isochorismate, D-mannuronic acid, biotin, and various nucleotide and glycosaminoglycan-related compounds. Conversely, 2,8-dihydroxycinnamic acid and 2,3-dihydroxycinnamic acid showed lower abundance at T2 than at T4. The exercise period (T2) was thus characterized by increased accumulation of tryptophan–kynurenine pathway metabolites, glycosaminoglycan degradation products, and aromatic compounds, with these metabolite levels decreasing during the subsequent post-washout (T3) and post-control (T4) periods.

### 3.5. SCFAs and Bile Acid Composition of the Feces

**Figure 3:**
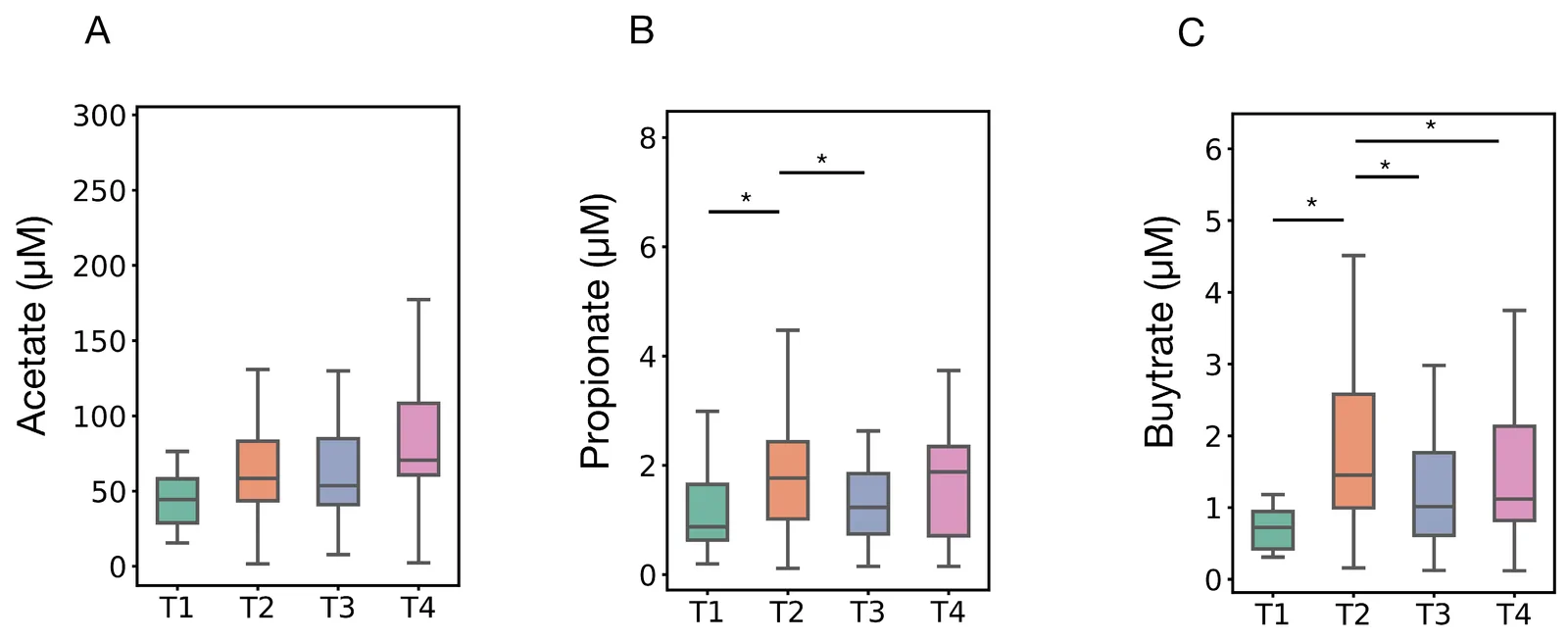
Short-chain fatty acid contents in the feces. Fecal concentrations of (**A**) acetate, (**B**) propionate, and (**C**) butyrate across T1 (baseline), T2 (post-exercise), T3 (post-washout), and T4 (post-control). Box plots display median, interquartile range (box), and range (whiskers). Horizontal lines indicate significant pairwise comparisons. * False discovery rate (FDR)-adjusted *q* < 0.05, linear mixed-effects models. *n* = 24.

Acetate levels showed no significant differences across the four time points (Figure 3A). Propionate concentrations were significantly higher at T2 than at T1 and T3 (Figure 3B). Butyrate concentrations were significantly higher at T2 than at T1, T3, and T4 (Figure 3C).

**Figure 4:**
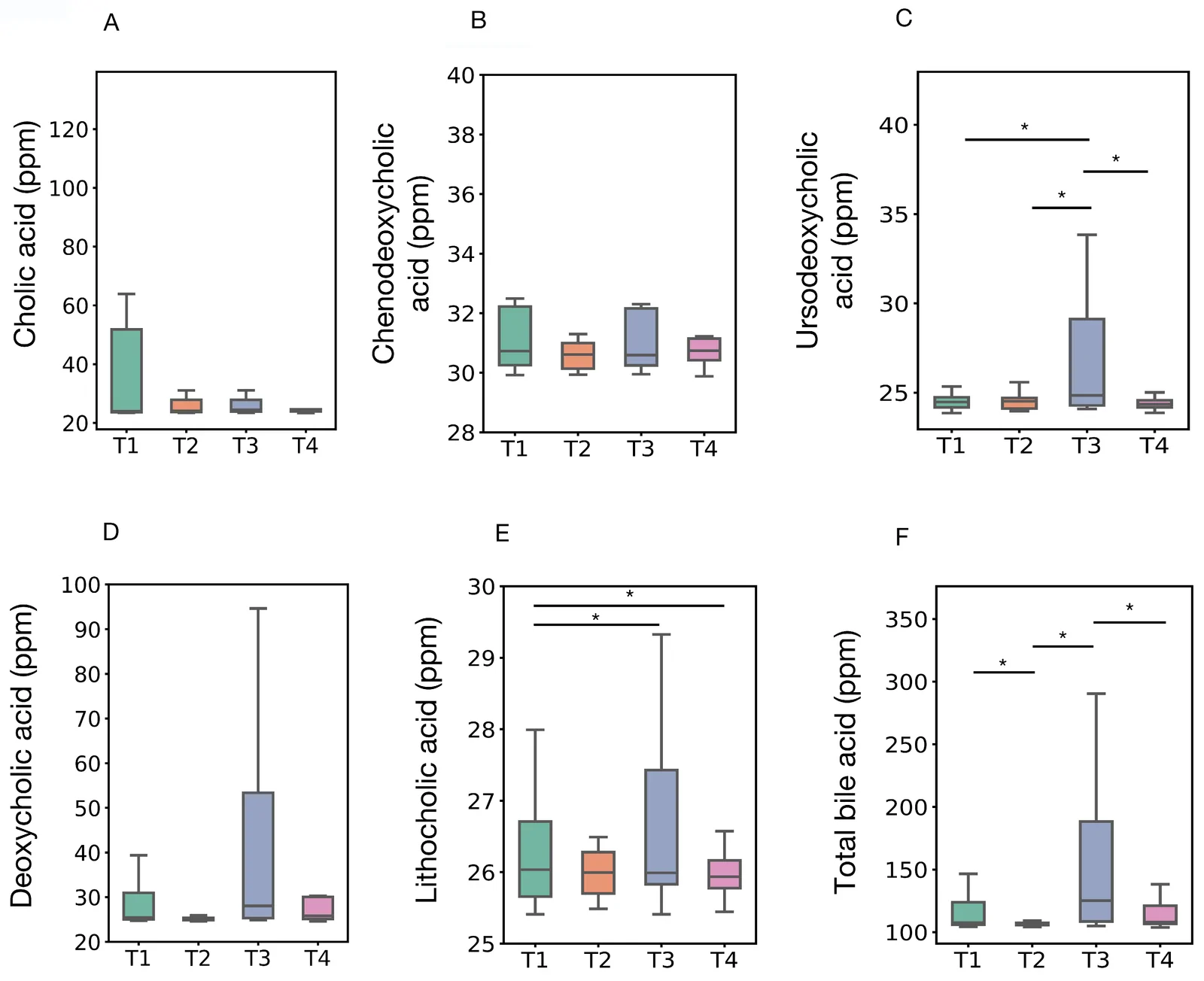
Bile acid content in the feces. Fecal concentrations of individual bile acids and total bile acids measured at baseline (T1), exercise intervention (T2), post-washout (T3), and post-control (T4): (**A**) cholic acid (CA), (**B**) chenodeoxycholic acid (CDCA), (**C**) ursodeoxycholic acid (UDCA), (**D**) deoxycholic acid (DCA), (**E**) lithocholic acid (LCA), and (**F**) total bile acids. Box plots show median (center line), interquartile range (box), and range (whiskers). Horizontal lines with asterisks indicate significant pairwise comparisons. * False discovery rate (FDR)-adjusted *q* < 0.05 (linear mixed-effects models with post hoc pairwise comparisons). *n* = 24 participants.

Among the five bile acids measured, only UDCA and LCA showed significant differences across time points. UDCA was significantly higher at T3 than at T1, T2, and T4 (Figure 4C). LCA was significantly higher at T3 than at T1, and significantly lower at T4 than at T1 (Figure 4E). The remaining bile acids (CA, CDCA, DCA) showed no significant differences among time points. Total bile acid concentrations were significantly higher at T1 than at T2, and higher at T3 than at both T2 and T4 (Figure 4F).

### 3.6. Causal Relationships Between Microbiota, Diet, and Metabolic Phenotypes by Tigramite Longitudinal Causal Analysis

**Figure 5:**
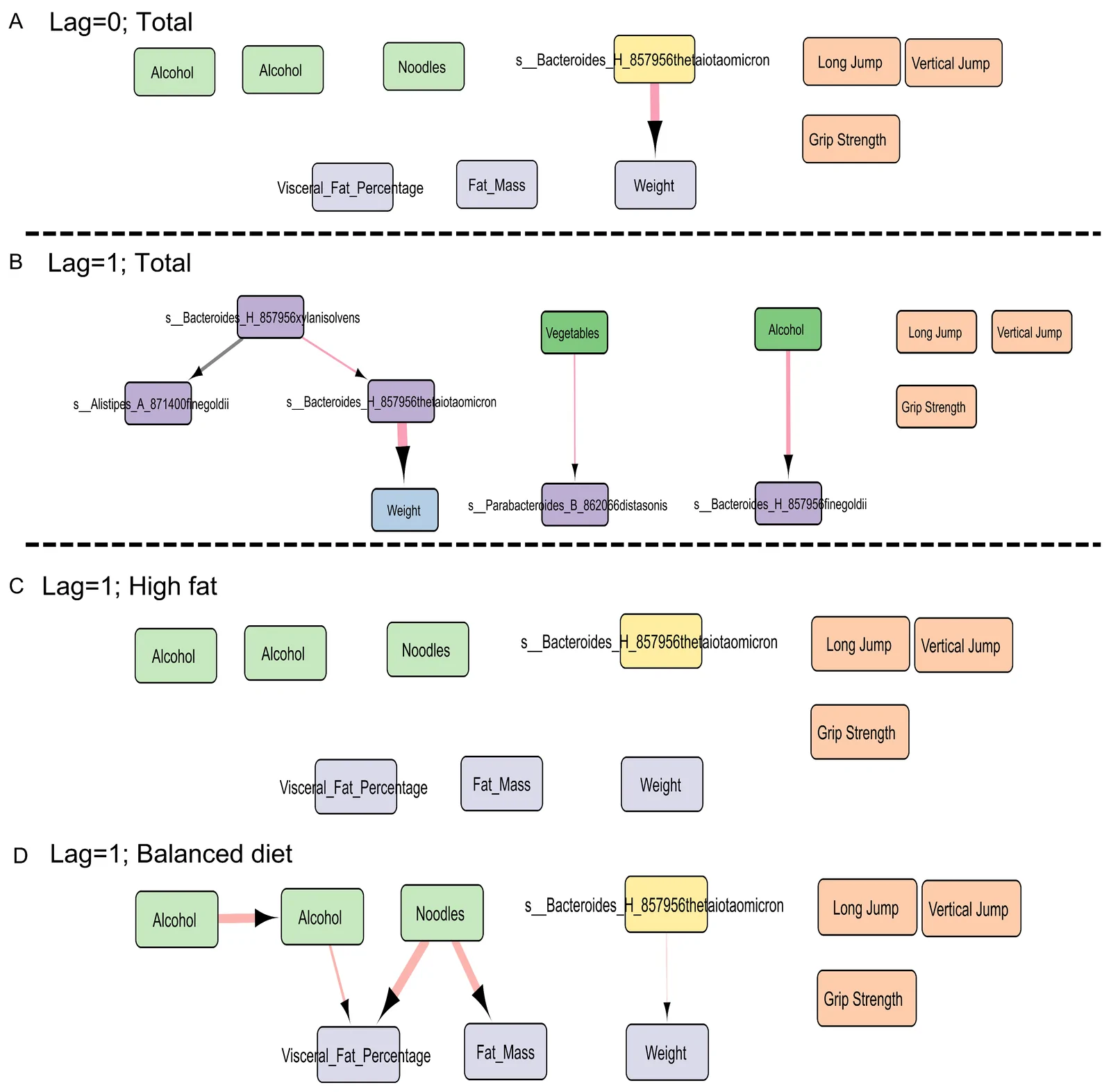
Temporal causal relationships among microbiota, diet, and host metabolism. Causal networks (Tigramite Peter and Clark Momentary Conditional Independence [PCMCI] algorithm) showing relationships among bacterial taxa, dietary intake, physical performance, and metabolic phenotypes: (**A**) contemporaneous associations (Lag 0, total cohort), (**B**) time-lagged causal effects (Lag 1, total cohort), (**C**) Lag 1 relationships in the balanced diet pattern group, and (**D**) Lag 1 relationships in Western-style diet pattern group. Nodes: bacterial taxa (yellow), dietary variables (green), body composition (purple), physical performance (orange). Arrows indicate causal direction from time t to t + 1. Dietary patterns modulate microbiota–host causal interactions, with multiple causal pathways detected in the balanced diet group but none in the high-fat Western-style diet group. *n* = 24.

We identified potential causal relationships between gut microbiota composition, dietary intake, and host metabolic phenotypes using the Tigramite framework (Figure 5), examining both contemporaneous relationships (Lag 0) and time-delayed causal effects (Lag 1), with stratification by dietary pattern. In the total participant analysis, *B. thetaiotaomicron* was positively associated with body weight both contemporaneously (Lag 0) and at Lag 1 (Figure 5A,B). At Lag 1, *B. xylanisolvens* positively predicted subsequent *B. thetaiotaomicron* abundance but negatively predicted *Alistipes finegoldii* abundance; vegetable consumption positively predicted *Parabacteroides distasonis* abundance; and alcohol consumption positively predicted *B. finegoldii* abundance (Figure 5B). Physical performance measures (long jump, vertical jump, grip strength) showed no causal relationships with any variable in any panel.

In the diet-stratified analysis at Lag 1, no significant causal relationships were detected in the WSD group (Figure 5C). In the BD group, higher alcohol consumption positively predicted subsequent alcohol consumption (autocorrelation) and visceral fat percentage; noodle consumption positively predicted both visceral fat percentage and fat mass; and *B. thetaiotaomicron* remained positively associated with body weight (Figure 5D).

### 3.7. RDA of Microbiota-Diet-Metabolite and Microbiota-Performance-Metabolite Relationships

**Figure 6:**
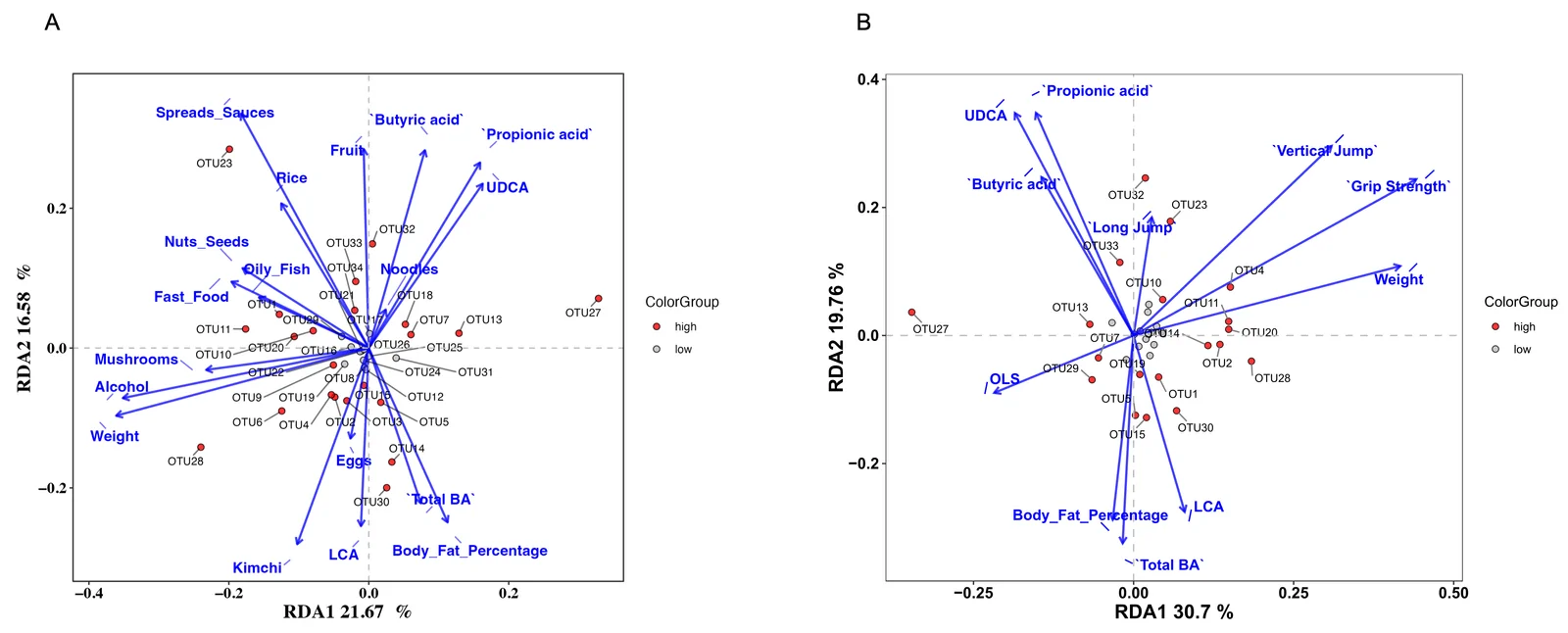
Redundancy analysis of microbiota-diet-metabolite and microbiota-performance-metabolite relationships. Redundancy analysis (RDA) biplots showing: (**A**) association of microbiota composition with dietary intake, short-chain fatty acids (SCFAs), and bile acids (38.25% variance explained; RDA1: 21.67%, RDA2: 16.58%); (**B**) associations of microbiota with physical performance measures, SCFAs, and bile acids (50.46% variance explained; RDA1: 30.7%, RDA2: 19.76%). Points: participants (red = Western-style diet, gray = balanced diet) and bacterial taxa (Operational Taxonomic Unit [OTU] numbers). OTU1, *Bacteroides xylanisolvens*; OTU2, *Parabacteroides johnsonii*; OTU4, *Phocaeicola vulgatus*; OTU5, *Rhodococcus erythropolis*; OTU6, *Bacteroides finegoldii*; OTU7, UMGS1071 sp.; OTU9, *Alistipes finegoldii;* OTU10, *Parabacteroides distasonis*; OTU11, *Bacteroides thetaiotaomicron*; OTU13, *Corynebacterium durum*; OTU14, *Alistipes putredinis*; OTU15, *Mediterraneibacter torques*; OTU18, *Lancefieldella* sp.; OTU19, *Ruthenibacterium lactatiformans*; OTU20, *Bacteroides stercoris*; OTU23, *Bacteroides fragilis*; OTU27, *Lachnoclostridium ammoniilytica*; OTU28, *Bacteroides uniformis*; OTU29, *Alitiscatomonas aceti*; OTU30, *Prevotella copri;* OTU32, *Adlercreutzia equolifaciens*; OTU33, *Otoolea saccharolyticum*; OTU34, *Fusobacterium ulcerans*. Arrows: environmental variables and metabolites (length and direction indicate association strength). Beneficial SCFAs clustered with fruit intake and long jump performance, while adiposity measures were associated with distinct microbial profiles. vegan package, 999 permutations, *p* < 0.05. *n* = 24.

To examine relationships between gut microbiota composition, dietary patterns, and metabolic outputs (SCFAs and bile acids), redundancy analysis (RDA) was performed. RDA revealed that dietary patterns and metabolites explained 38.25% of microbiota variance (RDA1: 21.67%, RDA2: 16.58%) (Figure 6A). Bacteroides fragilis (OTU23) was positioned in the upper-left region, in proximity to the fruit and butyric acid vectors. *Adlercreutzia equolifaciens* (OTU32), *Otoolea saccharolyticum* (OTU33), and *Fusobacterium ulcerans* (OTU34) were positioned near the plot’s vertical midline. *Bacteroides xylanisolvens* (OTU1), *Parabacteroides distasonis* (OTU10), and *Bacteroides thetaiotaomicron* (OTU11) were positioned in the upper-left region near the horizontal midline, closer to the oily fish, nuts, and fast-food vectors. *Lancefieldella* sp. (OTU18) was positioned near the noodles vector. *Parabacteroides johnsonii* (OTU2), *Phocaeicola vulgatus* (OTU4), *Bacteroides finegoldii* (OTU6), and *Bacteroides uniformis* (OTU28) were positioned in the lower-left region of the ordination, in the general direction of the kimchi vector.

RDA examining relationships between microbiota composition, physical performance measures, and metabolites explained 50.46% of microbiota variance (RDA1: 30.7%, RDA2: 19.76%) (Figure 6B). *Otoolea saccharolyticum* (OTU33) was positioned near the propionic acid, UDCA, and butyric acid vectors. *Bacteroides fragilis* (OTU23) was positioned near the long jump vector. *Phocaeicola vulgatus* (OTU4), *Bacteroides thetaiotaomicron* (OTU11), Parabacteroides distasonis (OTU10), and Bacteroides stercoris (OTU20) were positioned toward the vertical jump, grip strength, and weight vectors. *Bacteroides xylanisolvens* (OTU1), *Mediterraneibacter torques* (OTU15), *Rhodococcus erythropolis* (OTU5), *Prevotella copri* (OTU30), and *Ruthenibacterium lactatiformans* (OTU19) were positioned toward the body fat percentage, LCA, and total BA vectors.

## 4. Discussion

Most studies examining exercise effects on the gut microbiome rely on a single post-intervention assessment, which cannot distinguish a transient perturbation from a lasting shift, nor capture the trajectory by which any change resolves. This longitudinal study addressed this gap using a sequential exercise, washout, and control design with two independent post-intervention assessments, allowing us to evaluate both the immediate trajectory of change following cessation of exercise (T3) and the stability of a longer-term non-exercising state (T4), each relative to the pre-exercise baseline (T1). By tracking 24 adults over 24 weeks, we found that an 8-week self-directed exercise intervention period (T2) increased microbial diversity, was associated with higher abundance of several bacterial taxa, most notably *Ruminococcus gnavus*, along with *Bacteroides xylanisolvens* and *Phocaeicola vulgatus*, and enhanced propionate and butyrate production. These changes were not sustained: alpha diversity, several taxa, and SCFA concentrations declined by T3 and/or T4, with several measures showing no significant difference between T1 and T4, indicating that the microbial changes induced by exercise were reversible rather than persistent, consistent with the gut microbiome’s known plasticity in response to external stimuli [17,18].

Alpha diversity increased after exercise and declined by T4, consistent with studies showing higher microbial diversity in physically active individuals compared with sedentary controls [19]. Although the difference between baseline and post-exercise diversity was modest, the significant contrast between T2 and T4 underscores the transient nature of exercise-induced diversity changes, while the lack of a significant difference between T2 and T3 suggests this decline emerged gradually rather than immediately upon cessation of exercise. Beta diversity based on unweighted UniFrac distances showed no differences between T1 and T2, but significant differences emerged between T2 and both T3 and T4, with no difference between T1 and T4, supporting recovery toward the original microbial composition and demonstrating gut microbiome plasticity [18,20,21]. Bray–Curtis dissimilarity showed no significant differences across any pairwise comparison, suggesting the UniFrac-detected shift reflects turnover among lower-abundance, phylogenetically distinct taxa rather than the dominant community members, consistent with the fact that Bray–Curtis is driven primarily by shared taxon abundance while UniFrac incorporates phylogenetic relatedness [22]. Because Bray–Curtis did not diverge even during exercise itself, it could not further resolve whether T3/T4 represented a return to baseline or convergence toward a new state, leaving the UniFrac pattern as the primary evidence for reversibility in this study.

Exercise was associated with higher abundance of *Ruminococcus gnavus* (*p* < 0.001), along with *Bacteroides xylanisolvens* and *Phocaeicola vulgatus* at a less stringent threshold—taxa broadly implicated in complex carbohydrate fermentation [23,24]. *Bacteroides* species generate propionate via the succinate pathway [25], while *Ruminococcus gnavus* contributes to propionate through the propanediol pathway and supports butyrate production indirectly via cross-feeding with *Faecalibacterium prausnitzii* or *Eubacterium rectale* [24,26]. This pattern is consistent with the observed rise in fecal propionate and butyrate at T2, in line with previous studies linking exercise to enhanced SCFA production [27]. Redundancy analysis provided additional, independent support for a diet-microbiota-SCFA link: *Otoolea saccharolyticum* and *Bacteroides fragilis* were positioned in ordination space near the propionic acid, butyric acid, and UDCA vectors, suggesting these taxa may also contribute to SCFA and bile acid metabolism in this cohort. The elevated Bacteroidaceae/Lachnospiraceae ratio during exercise further suggests a shift toward greater carbohydrate fermentation capacity. Notably, while *Ruminococcus gnavus* has been linked to inflammation in clinical settings, its increase without adverse effects in this study suggests a beneficial metabolic role under moderate physiological demand [24,26], emphasizing exercise intensity as a critical determinant of microbial functional outcomes. Propionate improves glucose homeostasis and reduces hepatic lipogenesis through GPR43/AMPK signaling [28], while butyrate fuels colonocytes and modulates immune responses by inhibiting HDAC and activating G-protein-coupled receptors [29]. Stable acetate levels likely reflect production by a broad range of taxa, making it less sensitive to exercise-induced shifts, though this remains debated as some studies report exercise-induced acetate fluctuations [30].

Total fecal bile acids were lower during exercise than at baseline, washout, and control, suggesting altered host-microbiome handling of bile acids, possibly through changes in intestinal reabsorption, bacterial bile salt hydrolase (BSH) activity, or hepatic synthesis [31]. Notably, *Phocaeicola vulgatus* and *Ruminococcus gnavus*, both increased at T2, express BSH activity that deconjugates primary bile acids [32], offering a plausible taxon-specific mechanism for the altered bile acid pool observed during exercise, though this was not directly measured here. UDCA and LCA were both elevated at washout (T3) relative to baseline, but LCA was lower at T4, a pattern that does not follow a simple trajectory and may reflect shifts in bacterial 7α-dehydroxylation activity as the microbiome transitions away from its exercise-adapted state. UDCA has hepatoprotective effects, while LCA exerts condition-dependent effects [33]. As specific bile-acid-modifying taxa were not identified and fecal concentrations may not reflect circulating levels, the physiological relevance of these changes requires confirmation through targeted functional and circulating measurements.

Several additional metabolites showed altered abundance during exercise. Glycolate, D-mannuronic acid, N-acetyl-D-mannosamine, and D-glucosamine are amino-sugar and uronic acid derivatives generated during microbial degradation of host and dietary glycans; their altered abundance during exercise may reflect the same shift in carbohydrate fermentation capacity suggested by the elevated Bacteroidaceae/Lachnospiraceae ratio and increased abundance of glycan-degrading taxa such as *B. thetaiotaomicron* [34]. Sulfate showed a parallel pattern; among mucin-degrading gut bacteria, sulfate removal from mucin glycan structures has been observed specifically in *B. thetaiotaomicron* [35], one of the taxa found to increase at T2, suggesting this predicted rise in sulfate may reflect increased *B. thetaiotaomicron*-mediated desulfation of host mucin and other sulfated glycans such as heparan sulfate. Phenol and tyramine are products of bacterial fermentation of aromatic amino acids (tyrosine and phenylalanine); their exercise-associated changes may reflect shifts in proteolytic versus saccharolytic fermentation balance within the gut microbial community, a pattern previously linked to dietary substrate availability and microbial community composition. The dihydroxycinnamic acid isomers are microbial breakdown products of dietary polyphenols [36], and their altered abundance may reflect exercise-associated changes in polyphenol-metabolizing taxa or substrate turnover. Isochorismate and biotin are intermediates and end-products, respectively, of bacterial biosynthetic pathways (siderophore and vitamin B7 synthesis) [37]; their differential abundance may reflect broader shifts in microbial biosynthetic activity during exercise rather than a specific, targeted pathway effect. Deoxythymidine triphosphate (dTTP), a nucleotide precursor, may reflect changes in microbial DNA synthesis and turnover consistent with altered growth rates among exercise-responsive taxa. Collectively, these metabolite-level shifts point to a broad reorganization of microbial fermentative and biosynthetic activity during exercise, consistent with the taxonomic and functional pathway changes observed, though the physiological significance of each individual metabolite requires targeted validation.

Functional predictions suggested higher LPS biosynthesis and linoleic acid metabolism pathway abundance during exercise (T2) relative to washout and control. Rather than reflecting a favorable, anti-inflammatory shift, this pattern may instead reflect a transient, exercise-induced physiological stress response: acute bouts of exercise are known to transiently increase intestinal permeability and translocation of bacterial LPS into circulation, particularly under higher training loads, and this predicted rise in LPS biosynthesis capacity at T2 may parallel that phenomenon at the microbial level. The mechanistic basis for this predicted shift, whether related to changes in specific taxa, substrate availability, or dietary lipid intake, was not directly assessed in this study and requires further investigation. Metabolite predictions similarly suggested higher L-kynurenine during exercise, consistent with well-documented acute activation of the tryptophan–kynurenine pathway during exercise [38], driven by exercise-induced inflammatory and metabolic stress; this pathway is thought to normalize during recovery, consistent with the decline we observed in T3 and T4 [39,40]. Predicted glycosaminoglycan degradation products were also higher during exercise, potentially reflecting transient perturbation of the intestinal mucus layer under acute exercise stress [41], though the physiological relevance of these functional predictions requires confirmation through targeted metabolomics, as PICRUSt2 and COBRA Toolbox outputs represent inferred rather than directly measured functional capacity.

Temporal causal analysis revealed that *B. thetaiotaomicron* was positively associated with body weight contemporaneously (Lag 0) and at Lag 1 in the total cohort, and this Lag 1 association was also detected specifically within the BD subgroup, indicating a consistent and temporally robust relationship that was most readily detected under a balanced dietary pattern. *B. xylanisolvens* positively predicted subsequent *B. thetaiotaomicron* abundance but negatively predicted *Alistipes finegoldii* abundance, suggesting differential, taxon-specific temporal dynamics within the *Bacteroides*-associated community. Vegetable consumption positively predicted *Parabacteroides distasonis* abundance, and alcohol consumption positively predicted *Bacteroides finegoldii* abundance, indicating that dietary intake shaped specific taxa with a time delay.

Dietary pattern substantially modified these relationships: no significant time-lagged associations were detected in the WSD group, whereas the BD group showed a more elaborated causal network, with alcohol consumption positively predicting subsequent alcohol intake (autocorrelation) and visceral fat percentage, and noodle consumption positively predicting both visceral fat percentage and fat mass. This is consistent with reports that metabolic associations of *B. thetaiotaomicron* vary with dietary intake, including both positive and inverse relationships with adiposity depending on diet composition [42,43], and may reflect diet-dependent differences in bile acid or SCFA signaling through which this species influences host weight regulation. The detection of a more elaborated causal network specifically within the BD group suggests that a diet richer in fiber and unprocessed foods may create conditions, such as more stable substrate availability for fermentation, under which microbiota–host relationships become measurable, whereas a Western-style diet’s more heterogeneous and processed substrate profile may obscure or dampen these relationships rather than eliminate them. Dietary context should therefore be considered when interpreting longitudinal microbiome-metabolic relationships and designing microbiome-targeted interventions.

RDA provided a complementary, cross-sectional view of these relationships. *Bacteroides fragilis* clustered with fruit and butyric acid intake, consistent with this species’ fiber-fermenting capacity [44], while *B. xylanisolvens*, *P. distasonis*, and *B. thetaiotaomicron* clustered with a more heterogeneous set of foods (oily fish, nuts/seeds, fast food), reflecting that these species may have broad glycan-degrading versatility rather than a single substrate preference. Vertical jump, grip strength, and body weight clustered with the same taxa found to increase during exercise. Propionic acid, UDCA, and butyric acid formed a distinct cluster, positioned near Otoolea saccharolyticum, with long jump situated closer to this cluster than the other performance measures. Given the established role of propionate and butyrate in skeletal muscle energy metabolism via GPR41/43 signaling [30], this spatial pattern may reflect a link between fermentative capacity and explosive power output, though the association remains hypothesis-generating rather than confirmatory. Body fat percentage, LCA, and total bile acids clustered with *B. xylanisolvens*, *Mediterraneibacter torques*, *Rhodococcus erythropolis*, *Prevotella copri*, and *Ruthenibacterium lactatiformans*, though the specific contribution of each of these taxa to bile acid metabolism was not directly assessed and warrants further investigation. As Tigramite causal analysis found no direct temporal relationship between microbiota and any performance measure, these RDA associations most likely reflect a shared, parallel response to exercise and diet rather than a direct causal microbiota-performance pathway [45].

The major strengths of this study include a longitudinal design with two independent post-intervention assessments (washout and control), enabling evaluation of both the trajectory and durability of reversibility, alongside integration of compositional, functional, metabolite, and causal analyses with dietary stratification. Several limitations warrant acknowledgment. The exercise intervention was self-directed and non-standardized, with participants independently selecting exercise type, frequency, duration, and intensity. This may have supported adherence by aligning goals with individual preferences, reflecting a real-world approach to exercise promotion, but precluded characterization of training modality, cumulative load, and progressive overload, limiting mechanistic interpretation. Adherence relied on daily self-monitoring and weekly self-report rather than objective tracking, which may introduce reporting bias, and the physical performance measures used were general fitness indicators that may not have been optimally sensitive to individual training backgrounds. The small sample size (*n* = 24), and particularly the WSD subgroup (*n* = 7), likely limited power to detect subtle effects, including the null findings in that subgroup, especially given that the similarly modest BD group (*n* = 17) revealed a more elaborated causal network. The unequal sex distribution (19 males, 5 females) limited examination of sex-specific responses; future studies should ensure balanced representation. Given the sample size relative to the number of taxa tested, FDR correction was overly conservative for species-level analysis, so a stricter nominal threshold (*p* < 0.001) was used as a hypothesis-generating approach; taxa with effect sizes comparable to *Ruminococcus gnavus* that did not meet this threshold should be interpreted as suggestive rather than confirmed. RDA and Tigramite identify associative and temporally ordered relationships, not causation. Functional and metabolite predictions (PICRUSt2, COBRA Toolbox) require validation through shotgun metagenomics and targeted metabolomics. Dietary assessment relied on a self-reported SQFFQ, which is subject to reporting bias, including potential over- or under-reporting of energy and macronutrient intake, a well-documented limitation of food frequency questionnaires generally. Lifestyle variables (smoking, alcohol intake, sleep duration, water intake) and self-reported energy intake did not differ significantly across timepoints, and no participant reported probiotic use at any timepoint. However, relative dietary composition, assessed via PERMANOVA on Bray–Curtis distances, shifted significantly from T1 onward and did not revert by T3 or T4. Because this shift coincided with the exercise intervention period and persisted through washout and control, we cannot fully disentangle the specific contribution of exercise from concurrent dietary changes to the microbiome, metabolite, and functional shifts observed at T2; the Tigramite causal framework, which modeled dietary intake as a time-varying predictor across all four timepoints, partially addresses this by allowing diet-microbiota–host relationships to be assessed dynamically, and the finding that microbial community structure (via unweighted UniFrac) returned toward baseline by T4 despite the persistent dietary shift further suggests that diet composition alone does not fully account for the observed microbiome trajectory. This persistent dietary shift may also contribute to the sustained reductions in body fat mass and visceral fat percentage observed after the exercise intervention ended, independent of continued structured exercise. The mechanism underlying these sustained changes remains unclear and may reflect a lasting metabolic effect of the exercise intervention, untracked behavioral changes, or measurement variability, and warrants further investigation with more granular, objectively measured lifestyle tracking.

## 5. Conclusions

This 24-week longitudinal study demonstrates that exercise induces transient, largely reversible changes in the gut microbiome, including increased microbial diversity, higher abundance of *Ruminococcus gnavus* and related taxa, and elevated propionate and butyrate production, most of which declined during washout and control periods without sustained physical activity. Microbiota–host metabolic relationships were diet-dependent, with *Bacteroides thetaiotaomicron* consistently associated with body weight and additional causal pathways detected specifically under a balanced dietary pattern. These findings suggest that the metabolic benefits of exercise on the gut microbiome may depend on sustained activity and dietary context. The study population of healthy young adults from a single university may limit generalizability to other populations. Future research should incorporate larger, diverse cohorts with adequately powered dietary subgroups; standardized exercise protocols with varied intensities; shotgun metagenomics and targeted metabolomics for functional and metabolite validation; and plasma biomarkers to assess systemic metabolic and inflammatory effects.

## Data Availability

The original contributions presented in this study are included in the article/Supplementary Material. Further inquiries can be directed to the corresponding authors.

## Supplementary Materials

The following supporting information can be downloaded at: https://www.mdpi.com/article/10.3390/microorganisms14091867/s1, Figure S1. Study design flowchart; Figure S2. Longitudinal changes in relative dietary composition across the intervention periods; Figure S3. Longitudinal changes in body composition across the exercise, washout, and control periods; Figure S4. Physical performance measures across the exercise, washout, and control periods; Figure S5. Bray-Curtis dissimilarity of gut microbiota composition across the intervention periods; Figure S6. Cumulative bacterial composition at the genus level across the intervention periods; Figure S7. Differential abundance analysis for additional timepoint comparisons; Table S1. Participant characteristics at baseline and pre-control timepoints.

## Abbreviations

Abbreviation	Full term
AMPK	AMP-activated protein kinase
ASV	Amplicon sequence variant
BD	Balanced diet
BMI	Body mass index
CA	Cholic acid
CDCA	Chenodeoxycholic acid
CLR	Centered log-ratio
COBRA	COnstraint-Based Reconstruction and Analysis
DADA2	Divisive Amplicon Denoising Algorithm
DBP	Diastolic blood pressure
DCA	Deoxycholic acid
dTTP	Deoxythymidine triphosphate
ELSD	Evaporative light scattering detector
FDR	False discovery rate
FID	Flame ionization detector
FXR	Farnesoid X receptor
GC	Gas chromatography
GPR43	G protein-coupled receptor 43
HDAC	Histone deacetylase
HPLC	High-performance liquid chromatography
IRB	Institutional Review Board
KEGG	Kyoto Encyclopedia of Genes and Genomes
KoGES	Korean Genome and Epidemiology Study
LCA	Lithocholic acid
NGS	Next-generation sequencing
OLS	One-leg standing balance
OTU	Operational taxonomic unit
PCA	Principal component analysis
PCMCI	Peter and Clark Momentary Conditional Independence
PCoA	Principal coordinates analysis
PCR	Polymerase chain reaction
PERMANOVA	Permutational multivariate analysis of variance
PICRUSt2	Phylogenetic Investigation of Communities by Reconstruction of Unobserved States 2
QIIME2	Quantitative Insights Into Microbial Ecology 2
RDA	Redundancy analysis
SBP	Systolic blood pressure
SCFA	Short-chain fatty acid
SQFFQ	Semi-Quantitative Food Frequency Questionnaire
T1–T4	Study timepoints: baseline (T1), post-exercise (T2), post-washout (T3), post-control (T4)
TGR5	Takeda G-protein-coupled receptor 5
UDCA	Ursodeoxycholic acid
VMH	Virtual Metabolic Human
WSD	Western-style diet

## References

[1] Park S., Kang S., Kim D.S., Zhang T. (2022). Protection against Osteoarthritis Symptoms by Aerobic Exercise with a High-Protein Diet by Reducing Inflammation in a Testosterone-Deficient Animal Model. Life.

[2] Park S. (2021). Association between polygenetic risk scores related to sarcopenia risk and their interactions with regular exercise in a large cohort of Korean adults. Clin. Nutr..

[3] Jaremków A., Markiewicz-Górka I., Hajdusianek W., Czerwińska K., Gać P. (2023). The Relationship between Body Composition and Physical Activity Level in Students of Medical Faculties. J. Clin. Med..

[4] Hawley J.A., Forster S.C., Giles E.M. (2025). Exercise, the Gut Microbiome and Gastrointestinal Diseases: Therapeutic Impact and Molecular Mechanisms. Gastroenterology.

[5] Jyoti, Dey P. (2025). Mechanisms and implications of the gut microbial modulation of intestinal metabolic processes. NPJ Metab. Health Dis..

[6] Kang S., Jeong D.-Y., Seo J., Daily J.W., Park S. (2026). Microbiota-Mediated Bile Acid Metabolism as a Mechanistic Framework for Precision Nutrition in Gastrointestinal and Metabolic Diseases. Cells.

[7] Xu Y., Zhong F., Zheng X., Lai H.-Y., Wu C., Huang C. (2022). Disparity of Gut Microbiota Composition Among Elite Athletes and Young Adults With Different Physical Activity Independent of Dietary Status: A Matching Study. Front. Nutr..

[8] Aya V., Jimenez P., Muñoz E., Ramírez J.D. (2023). Effects of exercise and physical activity on gut microbiota composition and function in older adults: A systematic review. BMC Geriatr..

[9] Nicolas S., Dohm-Hansen S., Lavelle A., Bastiaanssen T.F.S., English J.A., Cryan J.F., Nolan Y.M. (2024). Exercise mitigates a gut microbiota-mediated reduction in adult hippocampal neurogenesis and associated behaviours in rats. Transl. Psychiatry.

[10] Li R., Zhang T., Cheng Z., Yao C., Meng X., Ma T. (2025). Advancements of physical exercise and intestinal microbiota and their potential mechanisms. Front. Microbiol..

[11] Park S., Kim K., Lee B.K., Ahn J. (2021). A Healthy Diet Rich in Calcium and Vitamin C Is Inversely Associated with Metabolic Syndrome Risk in Korean Adults from the KNHANES 2013–2017. Nutrients.

[12] Hur H.J., Yang H.J., Kim M.J., Jang H.J., Kim M.S., Park S. (2026). Precision nutrition for hypertension: Tea, coffee, antioxidant vitamins interactions with polygenic risk in multi-ethnic populations. Eur. J. Clin. Nutr..

[13] Wu X., Unno T., Kang S., Park S. (2021). A Korean-Style Balanced Diet Has a Potential Connection with Ruminococcaceae Enterotype and Reduction of Metabolic Syndrome Incidence in Korean Adults. Nutrients.

[14] Singh M.G., Wahengbam R. (2026). Optimization of DADA2 in QIIME2 for improving fidelity in 16S rRNA V4 amplicon data analysis. Biol. Methods Protoc..

[15] Kang R., Yu Z., Kim H., Seo J., Kim M., Park T. (2025). Manually weighted taxonomy classifiers improve species-specific rumen microbiome analysis compared to unweighted or average weighted taxonomy classifiers. Sci. Rep..

[16] Ruiz-Perez D., Gimon I., Sazal M., Mathee K., Narasimhan G. (2024). Unfolding and de-confounding: Biologically meaningful causal inference from longitudinal multi-omic networks using METALICA. mSystems.

[17] Varghese S., Rao S., Khattak A., Zamir F., Chaari A. (2024). Physical Exercise and the Gut Microbiome: A Bidirectional Relationship Influencing Health and Performance. Nutrients.

[18] Mohr A.E., Mach N., Pugh J., Grosicki G.J., Allen J.M., Karl J.P., Whisner C.M. (2025). Mechanisms underlying alterations of the gut microbiota by exercise and their role in shaping ecological resilience. FEMS Microbiol. Rev..

[19] Pérez-Prieto I., Plaza-Florido A., Ubago-Guisado E., Ortega F.B., Altmäe S. (2024). Physical activity, sedentary behavior and microbiome: A systematic review and meta-analysis. J. Sci. Med. Sport.

[20] Dziewiecka H., Buttar H.S., Kasperska A., Ostapiuk–Karolczuk J., Domagalska M., Cichoń J., Skarpańska-Stejnborn A. (2022). Physical activity induced alterations of gut microbiota in humans: A systematic review. BMC Sports Sci. Med. Rehabil..

[21] Elavsky S., Brabec M., Maly M., Knapova L., Kastovska B., Sebera M., Ely M., Jandackova V.K., Keller J., Pavel M. (2025). The temporal dynamics of the association between daily physical activity and life satisfaction. Ann. Behav. Med..

[22] Kers J.G., Saccenti E. (2022). The Power of Microbiome Studies: Some Considerations on Which Alpha and Beta Metrics to Use and How to Report Results. Front. Microbiol..

[23] Cheng J., Hu J., Geng F., Nie S. (2022). Bacteroides utilization for dietary polysaccharides and their beneficial effects on gut health. Food Sci. Hum. Wellness.

[24] Crost E.H., Coletto E., Bell A., Juge N. (2023). Ruminococcus gnavus: Friend or foe for human health. FEMS Microbiol. Rev..

[25] Wang X., Cai Z., Wang Q., Wu C., Sun Y., Wang Z., Xu X., Xue W., Cao Z., Zhang M. (2024). Bacteroides methylmalonyl-CoA mutase produces propionate that promotes intestinal goblet cell differentiation and homeostasis. Cell Host Microbe.

[26] Crost E.H., Tailford L.E., Monestier M., Swarbreck D., Henrissat B., Crossman L.C., Juge N. (2016). The mucin-degradation strategy of Ruminococcus gnavus: The importance of intramolecular trans-sialidases. Gut Microbes.

[27] Reljic D., Hermann H.J., Dieterich W., Neurath M.F., Zopf Y. (2025). Exercise improves gut microbial metabolites in an intensity-dependent manner: A pooled analysis of randomized controlled trials. Gut Microbes.

[28] Yoshida H., Ishii M., Akagawa M. (2019). Propionate suppresses hepatic gluconeogenesis via GPR43/AMPK signaling pathway. Arch. Biochem. Biophys..

[29] Siddiqui M.T., Cresci G.A.M. (2021). The Immunomodulatory Functions of Butyrate. J. Inflamm. Res..

[30] Liu X., Xu M., Wang H., Zhu L. (2025). Role and Mechanism of Short-Chain Fatty Acids in Skeletal Muscle Homeostasis and Exercise Performance. Nutrients.

[31] Zhang M., Xiao B., Chen X., Ou B., Wang S. (2024). Physical exercise plays a role in rebalancing the bile acids of the enterohepatic axis in non-alcoholic fatty liver disease. Acta Physiol..

[32] Chua H.H., Chen Y.H., Wu L.L., Yang H.C., Lin C.R., Chen H.L., Wu J.F., Chang M.H., Chen P.J., Ni Y.H. (2024). Antagonism Between Gut Ruminococcus gnavus and Akkermansia muciniphila Modulates the Progression of Chronic Hepatitis B. Cell Mol. Gastroenterol. Hepatol..

[33] Ward J.B.J., Lajczak N.K., Kelly O.B., O’Dwyer A.M., Giddam A.K., Gabhann J.N., Franco P., Tambuwala M.M., Jefferies C.A., Keely S. (2017). Ursodeoxycholic acid and lithocholic acid exert anti-inflammatory actions in the colon. Am. J. Physiol. Gastrointest. Liver Physiol..

[34] de Ram C., Berkhout M.D., Kozioł M., Blasco Matias L., Klostermann C., Pandeirada C.O., Boeren S., Ioannou A., Vincken J.P., Belzer C. (2026). Efficient mucin O-glycan degradation by specific mucin-degrading intestinal bacteria: Towards understanding enzyme-glycan interactions. Glycobiology.

[35] Dong J., Cui Y., Qu X. (2024). Metabolism mechanism of glycosaminoglycans by the gut microbiota: Bacteroides and lactic acid bacteria: A review. Carbohydr. Polym..

[36] Kiriyama Y., Tokumaru H., Sadamoto H., Kobayashi S., Nochi H. (2024). Effects of Phenolic Acids Produced from Food-Derived Flavonoids and Amino Acids by the Gut Microbiota on Health and Disease. Molecules.

[37] Wan Z., Zheng J., Zhu Z., Sang L., Zhu J., Luo S., Zhao Y., Wang R., Zhang Y., Hao K. (2022). Intermediate role of gut microbiota in vitamin B nutrition and its influences on human health. Front. Nutr..

[38] Agus A., Planchais J., Sokol H. (2018). Gut Microbiota Regulation of Tryptophan Metabolism in Health and Disease. Cell Host Microbe.

[39] Moffatt C.B., Plaman B.A., Rowe S.J., Caruso A., Early S.A., Ruiz N., Kahne D. (2025). Inhibiting Lipopolysaccharide Biogenesis: The More You Know the Further You Go. Ann. Rev. Biochem..

[40] Yoshida N., Yamashita T., Kishino S., Watanabe H., Sasaki K., Sasaki D., Tabata T., Sugiyama Y., Kitamura N., Saito Y. (2020). A possible beneficial effect of Bacteroides on faecal lipopolysaccharide activity and cardiovascular diseases. Sci. Rep..

[41] Rawat P.S., Seyed Hameed A.S., Meng X., Liu W. (2022). Utilization of glycosaminoglycans by the human gut microbiota: Participating bacteria and their enzymatic machineries. Gut Microbes.

[42] Wong J.P.H., Chillier N., Fischer-Stettler M., Zeeman S.C., Battin T.J., Persat A. (2024). Bacteroides thetaiotaomicron metabolic activity decreases with polysaccharide molecular weight. mBio.

[43] Xie S., Ma J., Lu Z. (2024). Bacteroides thetaiotaomicron enhances oxidative stress tolerance through rhamnose-dependent mechanisms. Front. Microbiol..

[44] Yang Y., Cao X., Kato N., Wang Y. (2027). Gut Bacteroides fragilis in health and diseases: An updated review. J. Future Foods.

[45] Fang J., Yan W., Sun X., Chen J. (2025). The role of exercise-induced short-chain fatty acids in the gut–muscle axis: Implications for sarcopenia prevention and therapy. Front. Microbiol..

