# Supplementary material for: Exercise Training Transiently Increases Gut Microbiota Diversity and Short-Chain Fatty Acid Production in a Diet-Dependent Manner in Healthy Adults
Source: Microorganisms. 2026 Aug 22;14(9):1867. doi: 10.3390/microorganisms14091867 (PMC13609704; doi:10.3390/microorganisms14091867)
Supplement: Supplementary file 1 [file microorganisms-14-01867-s001.zip › microorganisms-4483884-supplementary.pdf]

Supplementary Table

Table S1. Participant characteristics at baseline and pre-control timepoints.

Variables	T1 (Exercise; n=24)	T3 (No exercise; n=24)
Sex (M/F)	19/5	19/5
Age (years)	22.67±1.63*	23.01±1.69
Height (cm)	173.54±7.64	173.67±7.62
Weight (kg)	70.42±10.51	70.58±10.82
BMI (kg/m^2)	23.40±2.57	23.32±2.86
Systolic blood pressure (mmHg)	118±1.52	119±1.47
Diastolic blood pressure (mmHg)	78.3±6.8	78.6±5.9
Smoking status (Y/N)	8/16	9/15
Alcohol (Y/N)	16/8	17/7
Energy (Kcal/day)	3469±1434	2448±1082
Carbohydrate (En%)	69.2±11.6	66.3±10.0
Protein (En%)	13.3±3.7	12.7±3.2
Fat (En%)	16.7±6.1	18.9±6.3

T1 measurements were collected in Fall 2024, prior to the 8-week exercise intervention. T3 measurements were collected in early 2025, following the 8-week washout period and prior to the 8-week control period. The same 24 participants completed assessments at both timepoints. Approximately 4–5 months elapsed between T1 and T3, during which lifestyle factors and dietary habits may have changed naturally. Values are presented as number or mean ± SD. M, male; F, female; BMI, body mass index; Y, yes; N, no.

Supplementary figure legends

Figure S1. Study design flowchart

Flow diagram illustrating the sequence of the 8-week exercise intervention (Trial 1).

8-week washout and 8-week control period (Trial 2)

Figure S2. Longitudinal changes in relative dietary composition across the intervention periods. Principal coordinate analysis (PCoA) of Bray-Curtis distances calculated from relative Semi-Quantitative Food Frequency Questionnaire (SQFFQ) food-group frequencies at baseline (T1), post-exercise (T2), post-washout (T3), and post-control (T4). Repeated-measures PERMANOVA (9,999 intra-participant permutations) with Benjamini-Hochberg-corrected pairwise comparisons showed significant differences in dietary composition between T1 and T2, T1 and T3, and T1 and T4 ($q = 0.0012$), with no significant differences among T2, T3, and T4 ($q \geq 0.865$). Multivariate dispersion did not differ significantly between timepoints (PERMDISP, $q \geq 0.25$). n = 24 participants

Figure S3. Longitudinal changes in body composition across the exercise, washout, and control periods. (A) Body weight, (B) body fat mass, and (C) visceral fat percentage at baseline (T1), post-exercise (T2), post-washout (T3), and post-control (T4). Data are presented as box plots (median, interquartile range, and range) for n = 24 participants. Statistical comparisons were performed using linear mixed-effects models with FDR-corrected post-hoc pairwise comparisons (*$q < 0.05$, **$q < 0.01$, ***$q < 0.001$).

Figure S4. Physical performance measures across the exercise, washout, and control periods. (A) Grip strength, (B) long jump, (C) vertical jump, and (D) one-leg standing balance (OLS) at baseline (T1), post-exercise (T2), post-washout (T3), and post-control (T4). Data are presented as box plots (median, interquartile range, and range) for n = 24 participants. Statistical comparisons were performed using linear mixed-effects models with FDR-corrected post-hoc pairwise comparisons (*$q < 0.05$, **$q < 0.01$, ***$q < 0.001$).

Figure S5. Bray-Curtis dissimilarity of gut microbiota composition across the intervention periods. (A) Principal coordinates analysis (PCoA) of beta diversity based on Bray-Curtis dissimilarity, showing microbial community composition at baseline (T1), post-exercise (T2), post-washout (T3), and post-control (T4). (B) Within-subject Bray-Curtis dissimilarity for each pairwise timepoint comparison. Box plots show median (center line), interquartile range (box), and range (whiskers); individual points represent within-subject pairwise distances, connected by gray lines where applicable. PERMANOVA with FDR correction found no significant differences for any pairwise comparison (all $q \geq 0.63$). n = 24.

Figure S6. Cumulative bacterial composition at the genus level across the intervention periods. Relative abundance of the top bacterial genera at baseline (T1), post-exercise (T2), post-washout (T3), and post-control (T4). n = 24 participants.

Figure S7. Differential abundance analysis for additional timepoint comparisons. Volcano plots showing differential abundance of (A) bacterial species, (B) predicted functional pathways (PICRUSt2), and (C) predicted metabolites (COBRA Toolbox) for the T2 versus T1, T1 versus T3, and T1 versus T4 comparisons. No features reached statistical significance in these comparisons. The x-axis shows log2 fold change; the y-axis shows -log10(q-value). n = 24 participants.

Figure S1

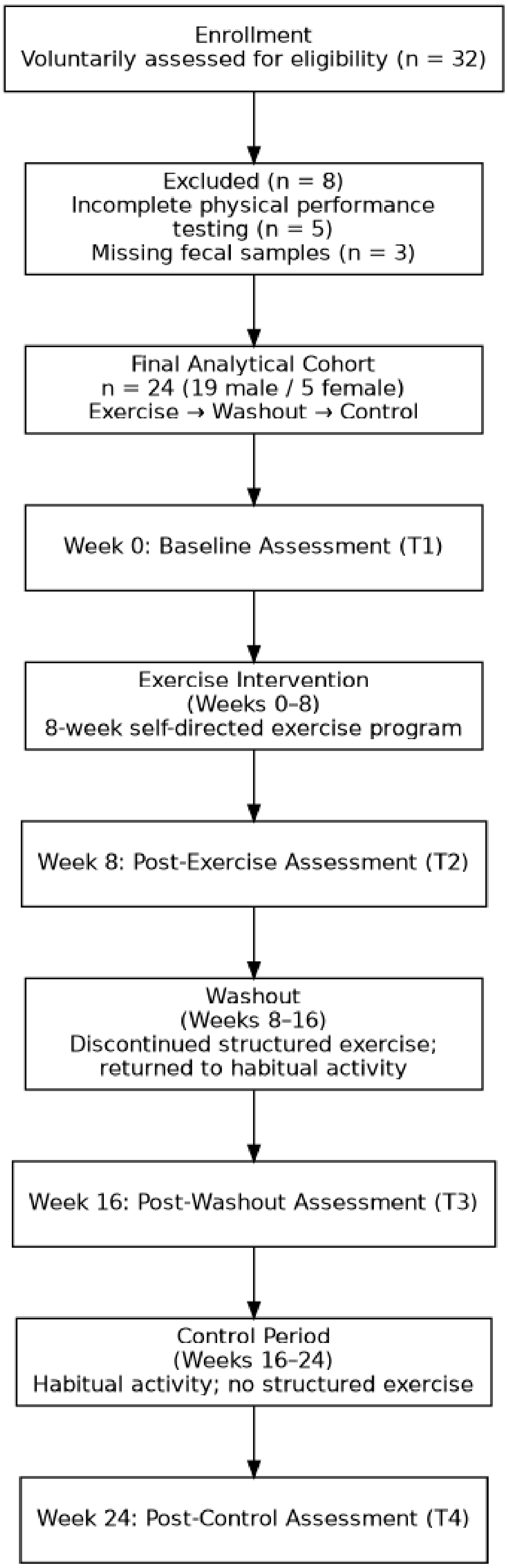
Enrollment
Voluntarily assessed for eligibility (n = 32)
Excluded (n = 8)
Incomplete physical performance testing (n = 5)
Missing fecal samples (n = 3)
Final Analytical Cohort
n = 24 (19 male / 5 female)
Exercise → Washout → Control
Week 0: Baseline Assessment (T1)
Exercise Intervention
(Weeks 0–8)
8-week self-directed exercise program
Week 8: Post-Exercise Assessment (T2)
Washout
(Weeks 8–16)
Discontinued structured exercise; returned to habitual activity
Week 16: Post-Washout Assessment (T3)
Control Period
(Weeks 16–24)
Habitual activity; no structured exercise
Week 24: Post-Control Assessment (T4)

Figure S2

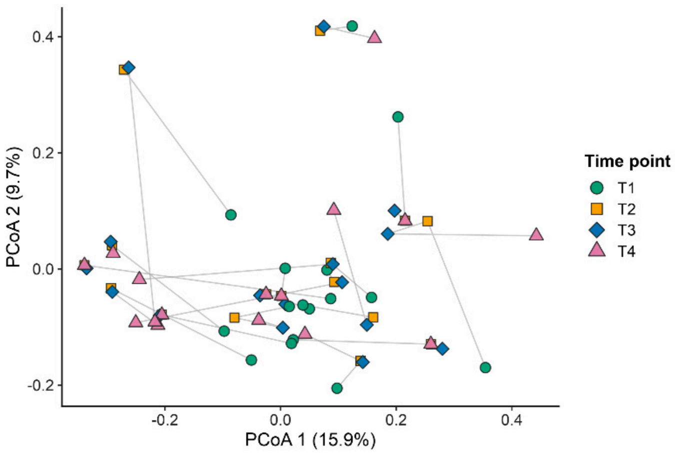

Figure S3

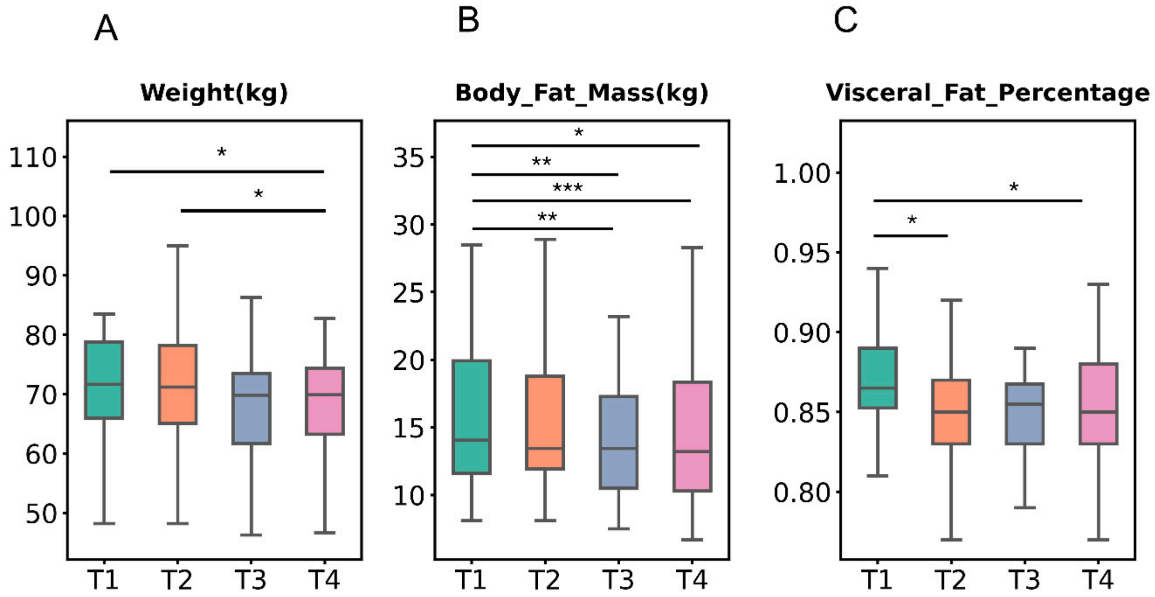
A
B
C
Weight(kg)
Body_Fat_Mass(kg)
Visceral_Fat_Percentage
110
100
90
80
70
60
50
35
30
25
20
15
10
1.00
0.95
0.90
0.85
0.80
*
**
***
T1
T2
T3
T4

Figure S4

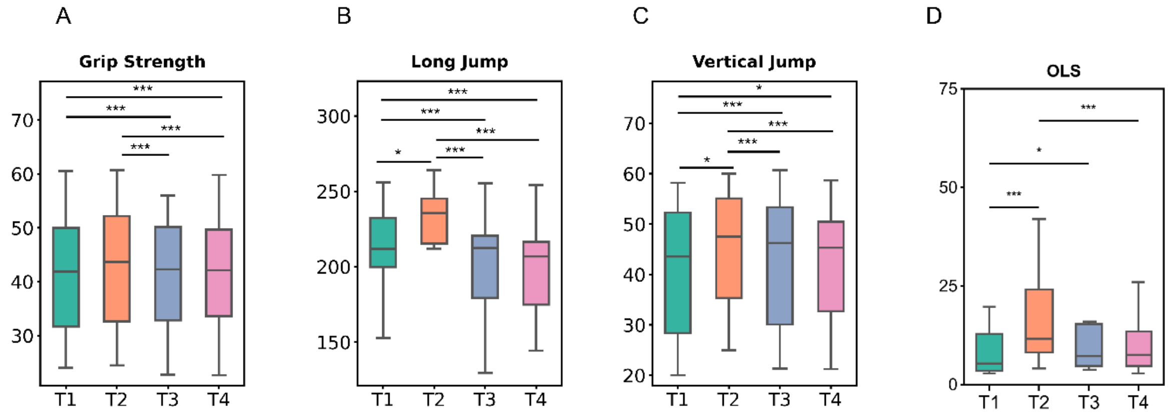

Figure S5

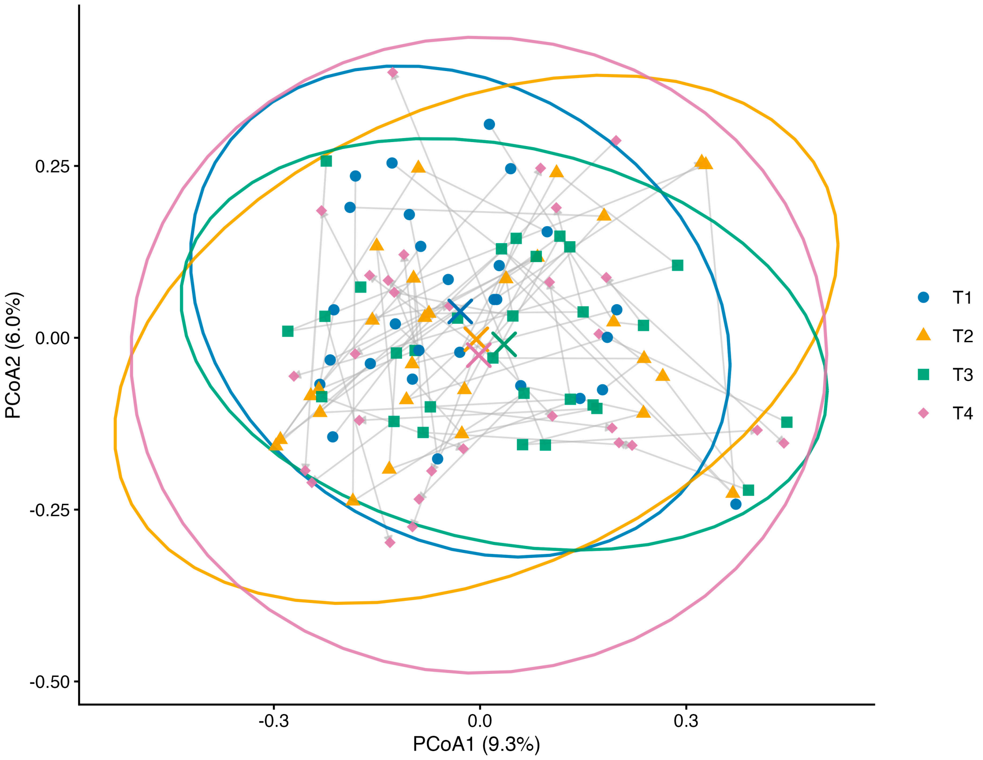

Figure S6.

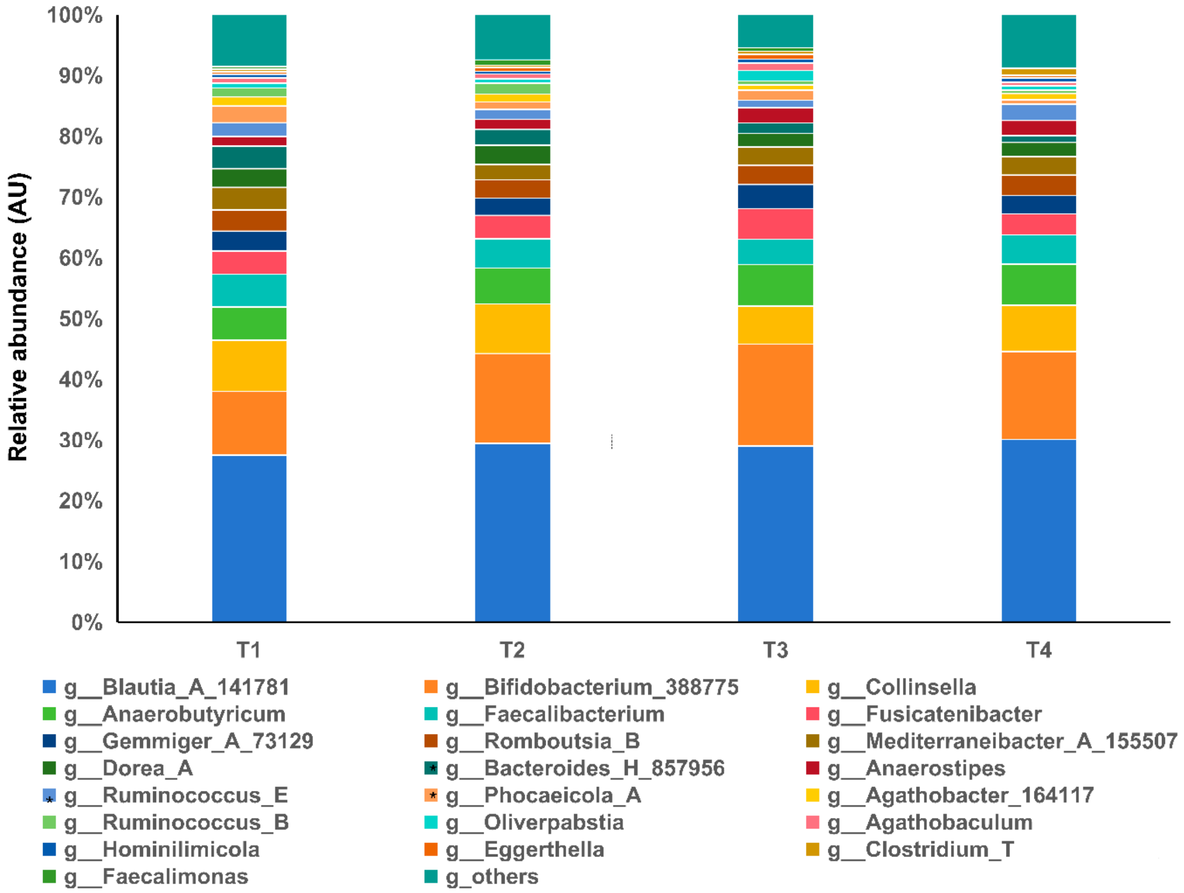

Relative abundance (AU)
100%
90%
80%
70%
60%
50%
40%
30%
20%
10%
0%
T1
T2
T3
T4
g__Blautia_A_141781
g__Bifidobacterium_388775
g__Collinsella
g__Anaerobutyricum
g__Faecalibacterium
g__Fusicatenibacter
g__Gemmiger_A_73129
g__Romboutsia_B
g__Mediterraneibacter_A_155507
g__Dorea_A
g__Bacteroides_H_857956
g__Anaerostipes
g__Ruminococcus_E
g__Phocaeicola_A
g__Agathobacter_164117
g__Ruminococcus_B
g__Oliverpabstia
g__Agathobaculum
g__Hominilimicola
g__Eggerthella
g__Clostridium_T
g__Faecalimonas
g_others

Figure S7.

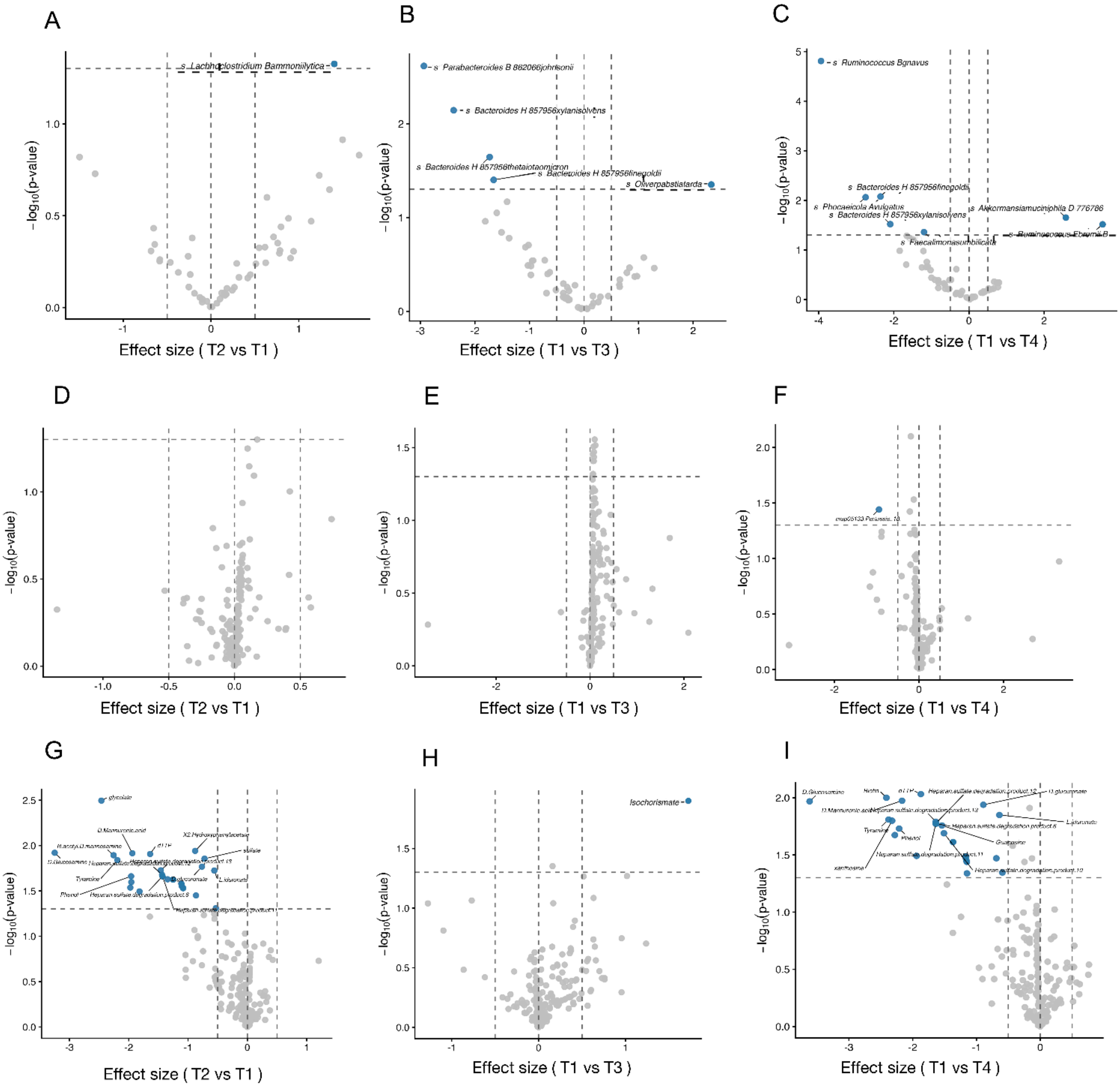
A
B
C
D
E
F
G
H
I
-log10(p-value)
Effect size (T2 vs T1)
Effect size (T1 vs T3)
Effect size (T1 vs T4)
s_Ruminococcus Bgnavus
s_Bacteroides H 857956xylanisolvens
s_Parabacteroides B 862066johnsonii
Isochorismate
